# Single-cell and spatial architecture of primary liver cancer

**DOI:** 10.1038/s42003-023-05455-0

**Published:** 2023-11-20

**Authors:** Pei-Yun Zhou, Cheng Zhou, Wei Gan, Zheng Tang, Bao-Ye Sun, Jin-Long Huang, Gao Liu, Wei-Ren Liu, Meng-Xin Tian, Xi-Fei Jiang, Han Wang, Chen-Yang Tao, Yuan Fang, Wei-Feng Qu, Run Huang, Gui-Qi Zhu, Cheng Huang, Xiu-Tao Fu, Zhen-Bin Ding, Qiang Gao, Jian Zhou, Ying-Hong Shi, Yong Yi, Jia Fan, Shuang-Jian Qiu

**Affiliations:** 1 Department of Liver Surgery and Transplantation, Liver Cancer Institute, Zhongshan Hospital, Fudan University, and Key Laboratory of Carcinogenesis and Cancer Invasion, Ministry of Education, Shanghai, 200032 China; 2 Shanghai Cancer Center, Fudan University, Shanghai, 200032 China

**Keywords:** Hepatocellular carcinoma, Cancer microenvironment

## Abstract

Primary liver cancer (PLC) poses a leading threat to human health, and its treatment options are limited. Meanwhile, the investigation of homogeneity and heterogeneity among PLCs remains challenging. Here, using single-cell RNA sequencing, spatial transcriptomic and bulk multi-omics, we elaborated a molecular architecture of 3 PLC types, namely hepatocellular carcinoma (HCC), intrahepatic cholangiocarcinoma (ICC) and combined hepatocellular-cholangiocarcinoma (CHC). Taking a high-resolution perspective, our observations revealed that CHC cells exhibit internally discordant phenotypes, whereas ICC and HCC exhibit distinct tumor-specific features. Specifically, ICC was found to be the primary source of cancer-associated fibroblasts, while HCC exhibited disrupted metabolism and greater individual heterogeneity of T cells. We further revealed a diversity of intermediate-state cells residing in the tumor-peritumor junctional zone, including a congregation of CPE^+^ intermediate-state endothelial cells (ECs), which harbored the molecular characteristics of tumor-associated ECs and normal ECs. This architecture offers insights into molecular characteristics of PLC microenvironment, and hints that the tumor-peritumor junctional zone could serve as a targeted region for precise therapeutical strategies.

## Abstract (web summary)

A comprehensive multi-omic, single-cell, and spatial analysis provides further insight into the major cell types present in three types of primary liver cancer.

## Introduction

Primary liver cancer (PLC), one of the leading causes of death among all cancers worldwide, mainly comprises heterogeneous hepatocellular carcinoma (HCC, 75 ~ 85%), intrahepatic cholangiocarcinoma (ICC, 10 ~ 15%), and combined hepatocellular-cholangiocarcinoma (CHC, 1 ~ 3%)^1–5^ with common and/or distinct molecular characteristics. Most of them are adenocarcinoma with inflammatory features (hepatitis virus infection and cholangitis), have common origins (hepatic progenitor cells) or conversions between mature hepatocytes and cholangiocytes. The sophistication of the molecular regulatory network and inadequate understanding of the relationship between PLC and the tumor microenvironment (TME) impede the exploration of its mechanism, drug access and therapy responses^6–14^. Recently, there has been a rising interest in the combination of immune checkpoint blockades (ICBs) and tyrosine kinase inhibitors (TKIs)^15,16^, which has been proved more effective for treating HCC and ICC than monotherapy^17–19^ but remains unsatisfactory with discordant responses. Targeting immune cells or stromal cells, by modulating their functionality, numbers, or subtype, is being explored as an effective therapeutic strategy against cancer. However, such an approach is faced with numerous challenges – not only because of TME’s pro-tumorigenic and anti-tumorigenic effects, but also the intralesional, intra-tumoral, inter-tumoral, individual heterogeneity, and other unknown key points (epigenetic, mutation, and metabolic variation, et al.)^20^. In particular, considering fresh sample requirements, CHC is rarely investigated in a high resolution due to its relatively low incidence and pre-operative diagnosis uncertainty^4^. Therefore, a thoroughly investigated landscape is urgently needed for understanding PLC.

**Fig. 1:**
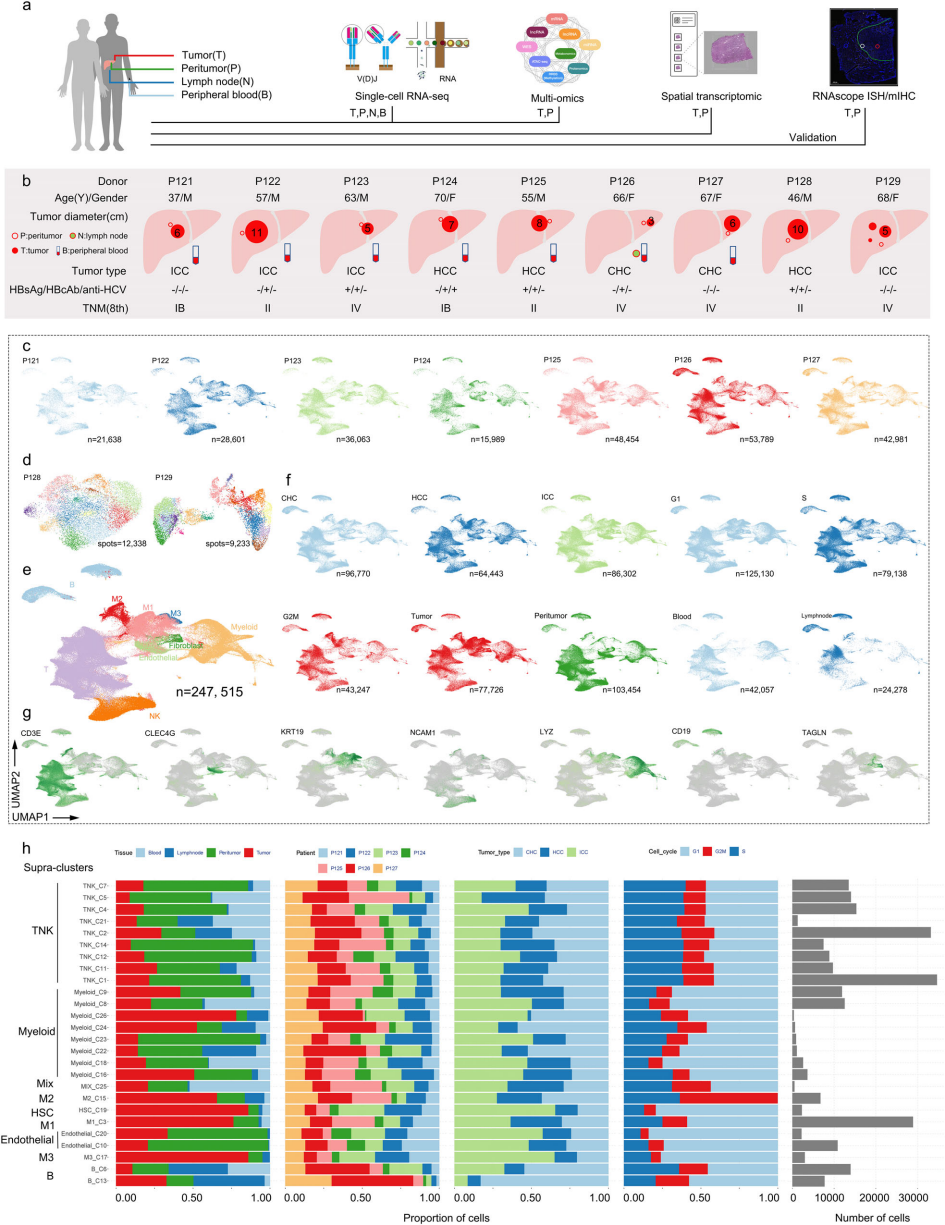
Molecular architecture of PLC. **a** Experimental workflow using scRNA-seq, multi-omics and ST, validated by RNAscope ISH and mIHC. **b** Demographic and clinical characteristics of 9 donors and related sample collection of tumor, peritumor, lymph node and peripheral blood (P121-P127 for scRNA-seq and multi-omics, and P128-P129 for ST). **c** UMAP distribution of single cells from 7 donors’ perioperative samples (P121-P127). **d** UMAP distribution of ST spots from 2 donors (P128-P129). **e** UMAP distribution of cell populations (247,515 cells from 22 peri-operative samples). T, T cells; NK, natural killer cells; B, B cells; myeloid, myeloid cells; endothelial, endothelial cells; fibroblast, fibroblast cells or hepatic stellate cells; M1, M2, M3, supra-clusters related to malignant cells. **f** UMAP distribution based on tumor type, cell cycle phase, tissue type. It shows an equilibrium distribution that was not affected by these factors. **g** UMAP distribution based on canonical marker gene of supra-clusters. **h** Bar plots of supra-clusters based on tissue type, patient, tumor type, cell cycle phase and cell number. ST, spatial transcriptomics; UMAP, uniform manifold approximation and projection; G1, gap 1 Phase; S, synthesis phase; G2M, gap 2 and mitotic phase. Mix, mixed and unassigned cells or clusters.

Single-cell RNA sequencing (scRNA-seq) has emerged as a potent approach to exploring cancer and its microenvironment in a single-cell resolution^21–26^ but with a deficiency in a morphological resolution for exploring solid tumors. Spatial transcriptomics (ST), a lately-developed high-throughput technology of RNA sequencing, overcomes such limitation with reliability in spot-cellular (10-200 cells) resolution^27–29^. However, both technologies are inadequate in the respects of epigenetics, proteomics and metabolomics. Thus, we reasoned that multi-omics with bulk-tissue resolution profiling might have the potential for addressing such deficiencies. Here, we present an elaborated landscape of PLC, simultaneously featuring HCC, ICC and CHC and covering a diversity of viral characteristics and tumor sizes. This endeavor offers an opportunity for a panoramic view of and parallel comparison among different PLC types rather than investigation in separation^21–23,25^. Multiscale integration of single-cell transcriptomics and immune repertoire, spot-cell ST and bulk-tissue multi-omics were incorporated to construct a valuable reference compendium for a comprehensive understanding of PLC. Multiplex fluorescent RNA in-situ hybridization (RNAscope ISH) and fluorescent multiplex immunohistochemistry (mIHC) were also employed to validate patterns of gene expression and tissue architecture (Fig. 1a).

## Results

### Multimodal landscape profiling of primary liver cancer

Single cells (SC) were isolated from a portion of paired fresh tumor, peritumor and peripheral blood from 7 donors with pathologically confirmed PLC (P121-P123 ICC, P124-P125 HCC, P126-P127 CHC), meanwhile a portion of metastatic lymph node was collected from P126. After one month, postoperative peripheral blood was also collected (P124, P125, P127, 2 samples from P123). A total of 27 samples were performed with scRNA and scV(D)J sequencing (Fig. 1a, b, Supplementary Data 1a). Internal validation tissues (another portions of tissues from SC cohort) and external validation tissues were collected to verify the difference discovered from SC data using RNAscope ISH. Detailed information of the profile is provided in Supplementary Table 1. After quality control and unsupervised processing with optimal settings (Supplementary Fig. 1a), the SC dataset comprises 289,156 high-fidelity sequenced cells (247,515 from 22 peri-operative samples) and 110,013 V(D)J-detected cells (SC immune repertoire, 91,152 TCRs and 18,861 BCRs). After the first clustering of 247,515 cells, there were 4 major cell populations comprised of 25 supra-clusters, including 3 supra-clusters related to malignant cells (M1, M2 and M3, marked with *GPC3*, *AKR1B10* and *KRT19*), 19 supra-clusters of immune cells (9 TNK supra-clusters comprised of T, NK and NKT cells, marked with *CD3*, *NACM1*; 8 myeloid supra-clusters comprised of monocyte, macrophage, and dendritic cells, marked with *LYZ*; and 2 B cell supra-clusters, marked with *CD19*), 1 supra-cluster of hepatic stellate cells (HSC, also known as fibroblast cells, marked with *TAGLN*), and 2 supra-clusters of endothelial cells (EC, marked with *CLEC4G*). Of these, 64,443 cells were collected from HCC, 86,302 from ICC, and 96,770 from CHC (Fig. 1c-g, Supplementary Fig. 1b). Normalized mutual information (NMI, 0-1) was employed to assess clustering robustness. We scored and applied regression to single-cell cycle phases. After calculation, the NMI score between before and after regression was 0.85, which implies a relatively small effect of cell-cycle heterogeneity, thus we opted not to correct it in the downstream analysis. Notably, every supra-cluster was derived from all donors, indicating that the clustering was driven by cell types rather than by batch effects (Fig. 1h). The second clustering was then conducted in these 4 major cell populations (25 supra-clusters) respectively to obtain corresponding sub-clusters with more precise assignment for further analysis.

**Fig. 2:**
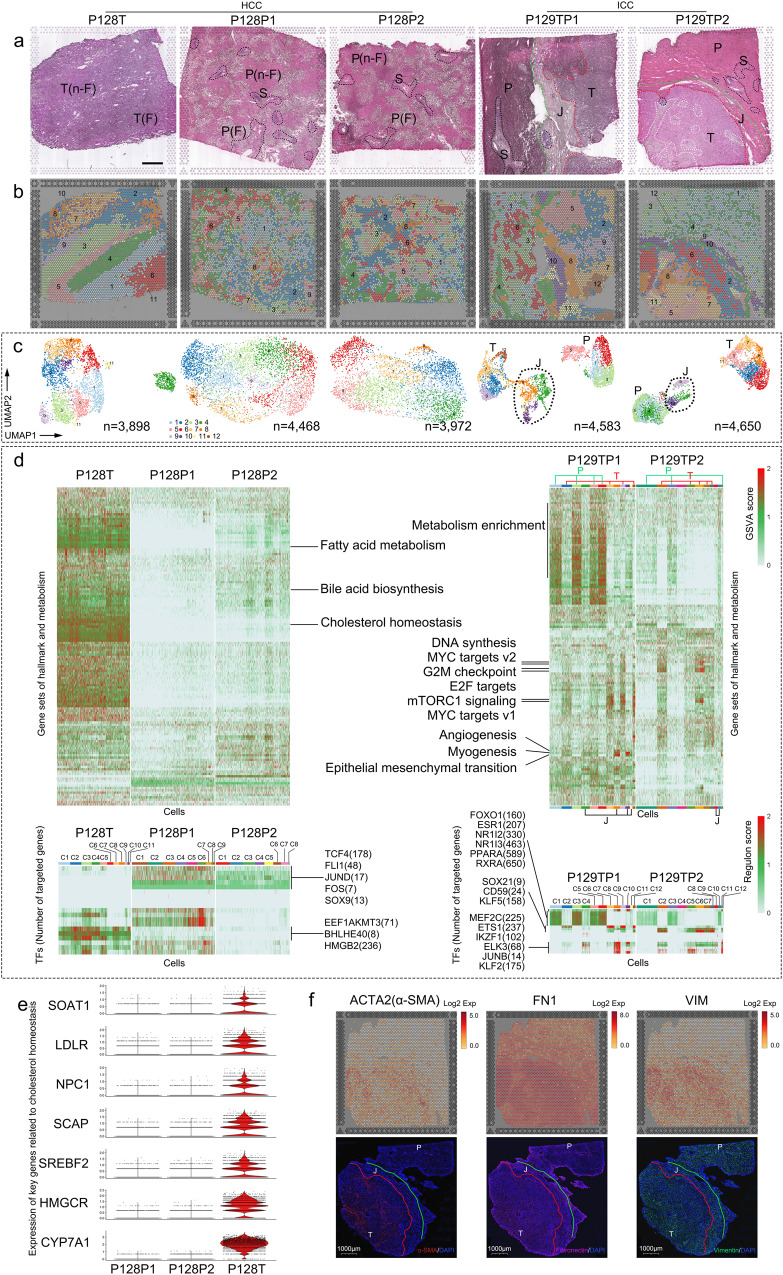
Spatial patterns of tumor, peritumor and tumor-peritumor junctional zones. **a** Annotated solid tissue cryosection on ST slices. T, tumor zone; P, peritumor zone; J, tumor-peritumor junctional zone (zone between blue and red dotted lines); S, stroma zone (black or white dotted lines); n-F, no-fatty infiltration region; F, fatty infiltration region; P128T, tumor tissue slice from P128; P128P1, peritumor tissue slice 1 from P128; P128P2, peritumor tissue slice 2 from P128; P129TP1, tumor-peritumor junctional zone tissue slice from P129 main lesion; P129TP2, tumor-peritumor junctional zone tissue slice from P129 sub-lesion. Scale bars, 1 mm. **b, c** Clustering (b) and UMAP distribution (c) of ST spots. Colors represent different clusters. **d** GSVA of P128 (upper left) and P129 (upper right). SCENIC analysis of P128 (lower left) and P129 (lower right). Light gray indicates lower enrichment and red indicates higher enrichment. **e** Expression of key genes related to cholesterol homeostasis in HCC ST slices. **f** Spatial expression pattern of marker genes related to fibroblast activation in P129TP2 ST slice (upper) and corresponding consecutive cryosection slice (lower). ACTA2, smooth muscle aortic alpha-actin; FN1, fibronectin-1; VIM, vimentin; Scale bars, 1000 µm.

To explore the spatial heterogeneity of PLCs, ST was performed with 5 extra tissues from 2 donors (P128 HCC, P129 ICC) (Fig. 1b, Supplementary Table 1, Supplementary Data 1a). A total of 21,571 ST spots were obtained from P128T, P128P1, P128P2, P129TP1 and P129TP2, at a median depth of 10,995 UMIs/spot and 3,092 genes/spot. The 3 slices of P128 were tumor-peritumor tissue separated, and 2 peritumor slices are consecutively collected, undergoing experimental repetition for a better data reliability (P128P1 and P128P2); the 2 slices of P129 came from where tumor-bordered peritumor, one slice from the main lesion (P129TP1), the other from the sub-lesion (P129TP2). Moreover, consecutive sections of ST cryo-tissues were also collected to verify the spatial expression pattern of ST data using fluorescent mIHC. Based on the histological structure of slices, we addressed 4 annotated spatial regions: tumor (T) zone, peritumor (P) zone, tumor-peritumor junctional (J) zone and stroma (S) zone. Slices from case P128 presented a feature of fatty infiltration, thus we differentiated non-fatty (n-F) areas from fatty (F) areas (Fig. 2a). To anchor dissolved single-cell clusters to their spatial locations by integrating the data from two modalities (scRNA-seq and ST), mutual nearest neighbor (MNN) and multimodal intersection analysis (MIA)^28^ were then performed. As expected, most non-parenchymal cells and immune cells were positioned in S or J zone (Supplementary Fig. 1c). Spatial spots were further deconvoluted based on the supra-clusters of scRNA-seq data^30^. We found that spots in T zone have a higher proportion of singlets, while spots in J and P zones showed more doublets that contain diverse cell types. Among 13985 predicted doublet spots (22249 assigned spots in total), those comprised of M3 and fibroblasts, M1 and fibroblasts, and M1 and endothelial cells are the majority, indicating the co-localization of these cells (Supplementary Fig. 1d, e).

To expand the borders of the molecular architecture of PLC, bulk tissue multi-omics were performed on 15 matched solid tissues (P121-P127), ranging from genome to metabolome: assay for transposase-accessible chromatin with high throughput sequencing (ATAC-seq) and reduced representation bisulfite sequencing (RRBS) in epigenomics, whole-exome sequencing (WES) in genomics, whole RNA-seq in transcriptomics, isobaric tandem mass tags (TMT)-based global proteomics, and LC-MS in metabolomics (Fig. 1a). From perspectives of different omics, the unsupervised clustering was conducted to explore heterogeneity at the tissue level. A chaotic distribution was found in epigenomics (peaks in the promoter region, methylation, miRNA7, and circRNA) and metabolomics, while a tissue-type-associated distribution was presented in mRNA, lncRNA and proteomics (Supplementary Fig. 2a, b), suggesting alterations in mRNA, lncRNA and proteomics have relatively larger effects on the phenotype. Analysis of somatic mutation found that the missense mutation was the most frequent variant, and that T127 presented a maximum number of single nucleotide variants (SNV) while N126 presented a minimum number of SNV and unsuspicious copy number variations (CNVs) compared with other samples (Supplementary Fig. 2c). Likewise, inferCNV predicted the P126N (i.e. N126 in bulk sequencing) had the minimum number of CNV using SC data compared with P121T (i.e. T126 in bulk sequencing) compared with P121T (i.e. T121 in bulk sequencing) (Supplementary Fig. 2d). Moreover, 8 distinct mutational signatures (S1-8) were identified by nonnegative matrix factorization. Of these, HCC-related S7 displayed a high cosine similarity (0.873) to Cosmic22 which related to exposure to aristolochic acid in previous HCC reports^31,32^ (Supplementary Fig. 2e). Together, these results suggest our bulk data is consistent with the SC data, and with known PLC genomic patterns to a certain degree.

### Tumor-peritumor junctional zone exhibits a complex pattern of spatial expression

To annotate the spatial expression pattern of regions, we conducted clustering analysis on each ST slice, finding that cluster spots of tumor tissue were more concentrated, while cluster spots of peritumor tissue appeared more disperse in terms of spatial distribution (Fig. 2a, b). This might result from the clonal evolutionary pattern of tumors^33^. Cluster spots in UMAP showed a tissue-associated distribution, in which spots of J zone were clustered and relatively separated from those of T and P zones (Fig. 2a-c). The corresponding relationship between clusters and annotated regions was also validated by principal component analysis, consistent with previous ST study^28^ (Supplementary Fig. 3a). Likewise, RNA velocity^34^ displayed a one-way progression from P to J to T in P129. These results suggested that J zone might have specific functional characteristics in cell developmental fate in contrast with T and P zones.

By gene set variation analysis (GSVA), 2 consecutive slices of peritumor tissue (P128P1 and P128P2) showed similar features in the enrichment of hallmark and metabolic gene sets. As expected, their enrichment was weaker than that in the slice of matched tumor tissue (P128T, Fig. 2d). For instance, gene sets including fatty acid metabolism, bile acid biosynthesis and cholesterol homeostasis were distinct features of P128T slice, consistent with a previous report on HCC^35^. Since disrupted cholesterol homeostasis has been reported as an aggressive characteristic of HCC with poor prognosis. We investigated 7 key genes related to cholesterol homeostasis, and all of them showed significant upregulation in P128T in comparison with the genes in paired peritumor tissues (Fig. 2e). Regarding the enrichment of gene sets in P129TP slices (ICC), it closely related to annotated regions, and clusters from same P or T zone shared similar characteristics internally. T zone showed the enrichment of mTORC1 signaling, MYC targets, G2M checkpoint, E2F targets, and DNA synthesis, which related to cell proliferation or oncogenesis; P zone showed the enrichment of gene sets in abnormal metabolism, such as bile acid biosynthesis and cholesterol metabolism, indicating cells bordered tumor might be affected by cells in T zone and orchestrate with them. Of note, clusters in J zone (C4, C8, C10, C11 in P129TP1; C9, C10 in P129TP2) exhibited a higher diversity than those in T or P zone. C8 and C10 in P129TP1 were specifically enriched with angiogenesis, myogenesis, and epithelial-mesenchymal transition (EMT), while the rest clusters presented relatively modest characteristics (Fig. 2d). Furthermore, fluorescent mIHC was conducted to validate our findings at a protein level using consecutive cryosection. It suggests the spatial pattern of 3 selected markers (related to fibroblast activation) in consecutive cryo-section is consistent with the spatial expression pattern in ST slices (Fig. 2f). These results support the ability to identify functional phenotypes of spatial regions based on ST data.

To further validate the spatial pattern of gene set enrichment, we applied single-cell regulatory network inference and clustering (SCENIC) to assessing the activity of gene sets of cells regulated by transcription factors (TFs), and we found it was consistent with the results of gene set enrichment. Two consecutive slices of peritumor tissue (P128P1 and P128P2) showed a similar pattern of transcriptional regulation (*TCF4, FLI1*, *JUND*, *FOS* and *SOX9*). *EEF1AKMT3*, *BHLHE40*, and *HMGB2* were activated in 11 tumor-related clusters of P128T. *HMGB2* was reported to be a pro-inflammatory DNA binding factor and related to cholesterol efflux^36^ and poor cancer prognosis^37^, consistent with our above-described abnormal cholesterol homeostasis in P128T. Likewise, C8 from J zone in P129TP1 presented active regulons regulated by *MEF2C*, *ETS1*, *IKZF1*, *JUNB ELK3* and *KLF2*. These TFs were associated with fibroblastic assembly, in line with the large amount of collagen fibers aggregated in J zone, while the rest clusters in J zone presented a transcriptional activity with less active TFs (Fig. 2d). Together, these results suggest J zone exhibited a relatively complex pattern of spatial expression compared with T or P zone. ST enables a stereoscopic investigation into the difference in phenotypes between HCC and ICC sample.

### CHC cells present internally discordant phenotypes while HCC and ICC have tumor-specific features

**Fig. 3:**
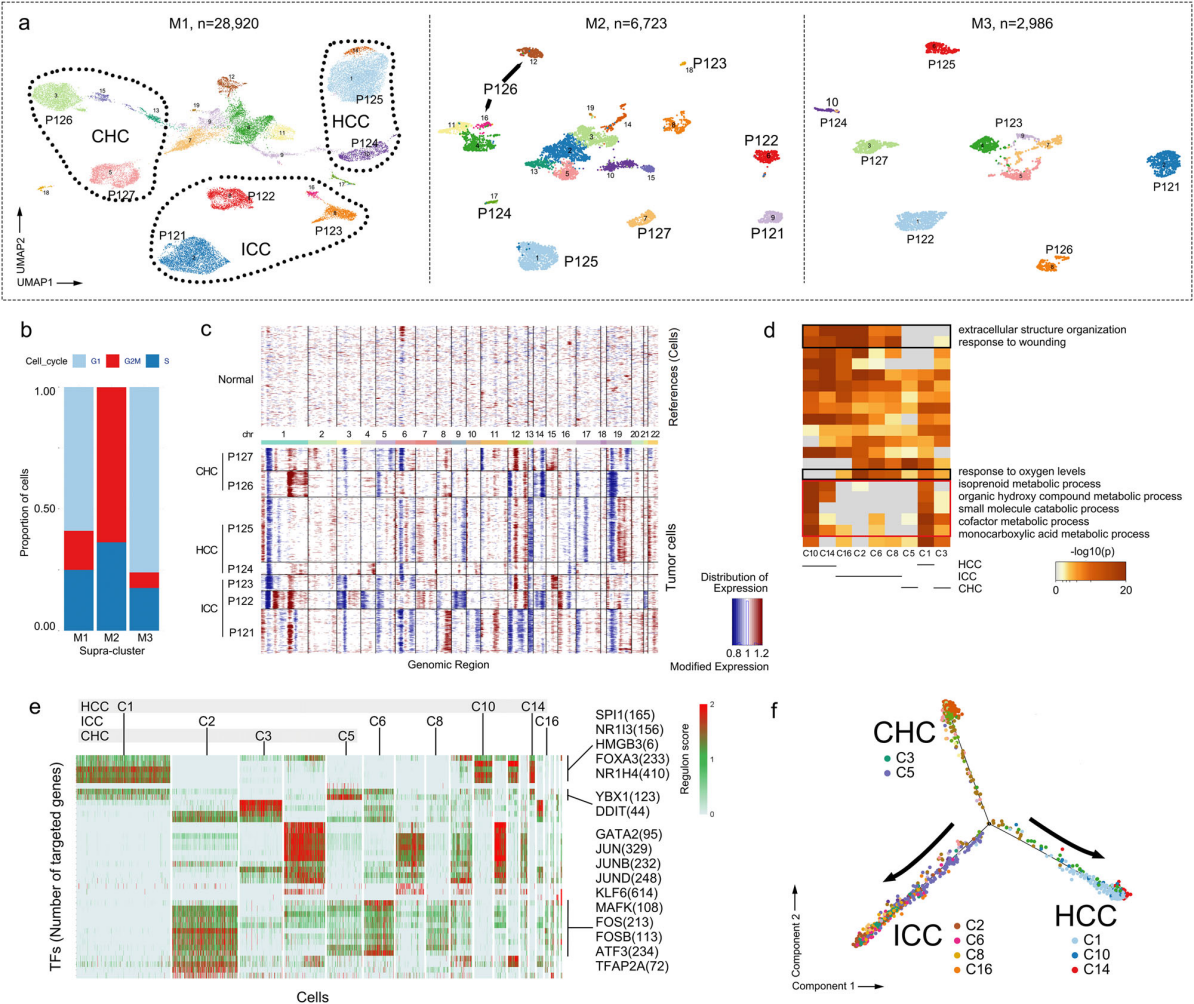
Distinct phenotypes of PLC’s malignant cells at a single-cell level. **a** UMAP distribution of M1, M2 and M3. Clustered cells present a distinctive patient-specific way. **b** Bar plot of cell cycle phase in terms of M1, M2 and M3. **c** Identification of somatic chromosomal CNV based on scRNA-seq data of patient-specific malignant sub-clusters from M1. **d** Metascape pathway enrichment based on malignant sub-clusters from M1. black frame, relatively enriched pathways in ICC sub-clusters; white frame, relatively enriched pathways in HCC sub-clusters. **e** SCENIC analysis of malignant sub-clusters from M1. **f** Pseudotime trajectory of malignant sub-clusters from M1.

The cohort covering three types of PLC enabled parallel analysis among them. Three supra-clusters involving malignant cells from the first clustering were labeled as M1 (C3, 28,920 cells), M2 (C12, 6723 cells), and M3 (C17, 2986 cells) (Fig. 3a). We observed significant heterogeneity among M1, M2 and M3, in which M2 showed higher proliferation of G2M or S phase than M1 and M3 (Fig. 3b). The heterogeneity was also validated by the specific high expression of *MKI67* in M2 (Supplementary Fig. 3b). Therefore, their downstream analysis was performed separately. The heterogeneity also existed at both intertumoral and individual levels. Through the second clustering, M1 was distinctly separated into 19 sub-clusters in UMAP, 9 of which clustered in a patient-specific way, and sub-clusters from M2 and M3 likewise (Fig. 3a, Supplementary Fig. 3c). Cells from these patient-specific sub-clusters mostly originated from tumor tissues, suggesting that these sub-clusters were mainly composed of malignant cells. Despite there were 3 sub-clusters (C4, C11, and C12) of M1 derived from peritumor tissues, but they were identified as mixed cell sub-clusters which presented a concentrated distribution in UMAP, and we did not observe sub-clusters of normal hepatocytes and cholangiocytes from the peritumor tissues in our PLC cohort (Supplementary Fig. 3c), in agreement with a previous study on HCC^38^. These mixed cell sub-clusters occur, likely due to limitations of current knowledge and technology. In addition, we applied inferCNV to identify somatic chromosomal CNV based on our scRNA-seq data of 9 patient-specific sub-clusters mentioned above, finding that cells from different patients varied in deletions and amplifications of chromosome. Our ICC profile displayed the particular CNV loss of chromosome 3 compatible with prior study on ICC^10^ (Fig. 3c).

InferCNV also helped to differentiate malignant cells from non-malignant cells, enabling the targeted analysis of 9 malignant sub-clusters from the second clustering of M1. Next, we separated these heterogeneous sub-clusters based on their types of PLC, finding the enrichment of abnormal metabolic processes in HCC sub-clusters (C1, C10, and C14), such as metabolism of isoprenoid, organic hydroxy compound, and monocarboxylic acid metabolism. In contrast, cells from ICC sub-clusters (C2, C6, C8, and C16) manifested the enrichment of abnormal oxygen metabolism and EMT (Fig. 3d). These results were largely consistent with the spatial expression pattern that we described above. Further, we employed SCENIC to predict transcription patterns of tumor cells, finding a series of key TFs of HCC tumorigenesis, such as TFs in regulation of cell dedifferentiation (*SPI1, HMGB3,* and *YBX1*) and in abnormal bile acid metabolism (*NR1H4, NR1I3, FOXA3* and *DDIT3*). Meanwhile, ICC sub-clusters presented a different pattern featuring *JUN, JUNB, JUND, FOS, FOSB, GATA2, KLF6, MAFK, ATF3*, and *TFAP2A*, the first 5 of which were enriched in AP-1 transcription factor family that may be related to epigenetic disruption and matrix formation^39,40^(Fig. 3e). Two CHC sub-clusters (C3 and C5) had a distinctive regulatory pattern compared with those from HCC and ICC. The gene set enrichment of CHC sub-clusters (e.g., abnormal monocarboxylic acid metabolism and response to oxygen levels) involved that of both HCC and ICC sub-clusters (Fig. 3d), while CHC sub-clusters in SCENIC displayed active regulons regulated by TFs that are different from those in HCC and ICC. Notably, the difference in TFs also remained between 2 CHC sub-clusters (C3 and C5) (Fig. 3e). It implies that CHC malignant cells might have internally discordant phenotypes, different from HCC and ICC malignant cells which have relatively consistent phenotypes respectively, partly due to the difference in clonal origins^4^.

Next, we investigated known driver genes based on bulk somatic mutations, obtaining a total of 91 oncogenes (Supplementary Fig. 3d). The expression of oncogenes in the sub-clusters of M1 were also detected in scRNA-seq. For instance, bulk-detected *KRAS* (T121), *JAK1* (T122), and *IDH1* (T123) were found in corresponding ICC-specific sub-clusters (C2, C6 and C16), and tumor suppressor genes such as *ARID1A* (T124) and *SMARCA4* (N126) were found transcriptionally inactive in corresponding sub-clusters^41–43^ (Supplementary Fig. 3e). Metabolic process of monocarboxylic acid was discovered in bulk HCC samples (ATAC-seq, mRNA-seq, and proteomics), consistent with HCC sub-clusters (C1, C10, and C14). Extracellular structure organization was also found a significant enrichment in ICC and CHC bulk tissues, compared to that in HCC (Supplementary Fig. 3f). Furthermore, CibersortX algorithm was employed to predict relative proportions of each SC supra-clusters in the bulk RNA-seq data. Malignant proportion from ICC bulk tissues (T121-T123) were mainly predicted in supra-cluster M3, and its counterpart from HCC (T124-T125) mainly in supra-cluster M1. Of note, CHC bulk tissues (T126-T127) presented a mixed proportion, validating that CHC had combined characteristics from ICC and HCC (Supplementary Fig. 3g). In the pseudotime trajectory, HCC and ICC were located at opposite ends of the trajectory, while CHC were partly located in the third end, and partly in the ICC end (Fig. 3f). Together, these results suggest that HCC and ICC have distinct phenotypes respectively. ICC features the enrichment of epithelial-mesenchymal transition, while HCC features metabolic abnormalities (i.g. monocarboxylic acid), and CHC presents internally discordant phenotypes compared to the other 2 PLC types.

### Cancer-associated fibroblasts harbor a tumor-type-specific feature

**Fig. 4:**
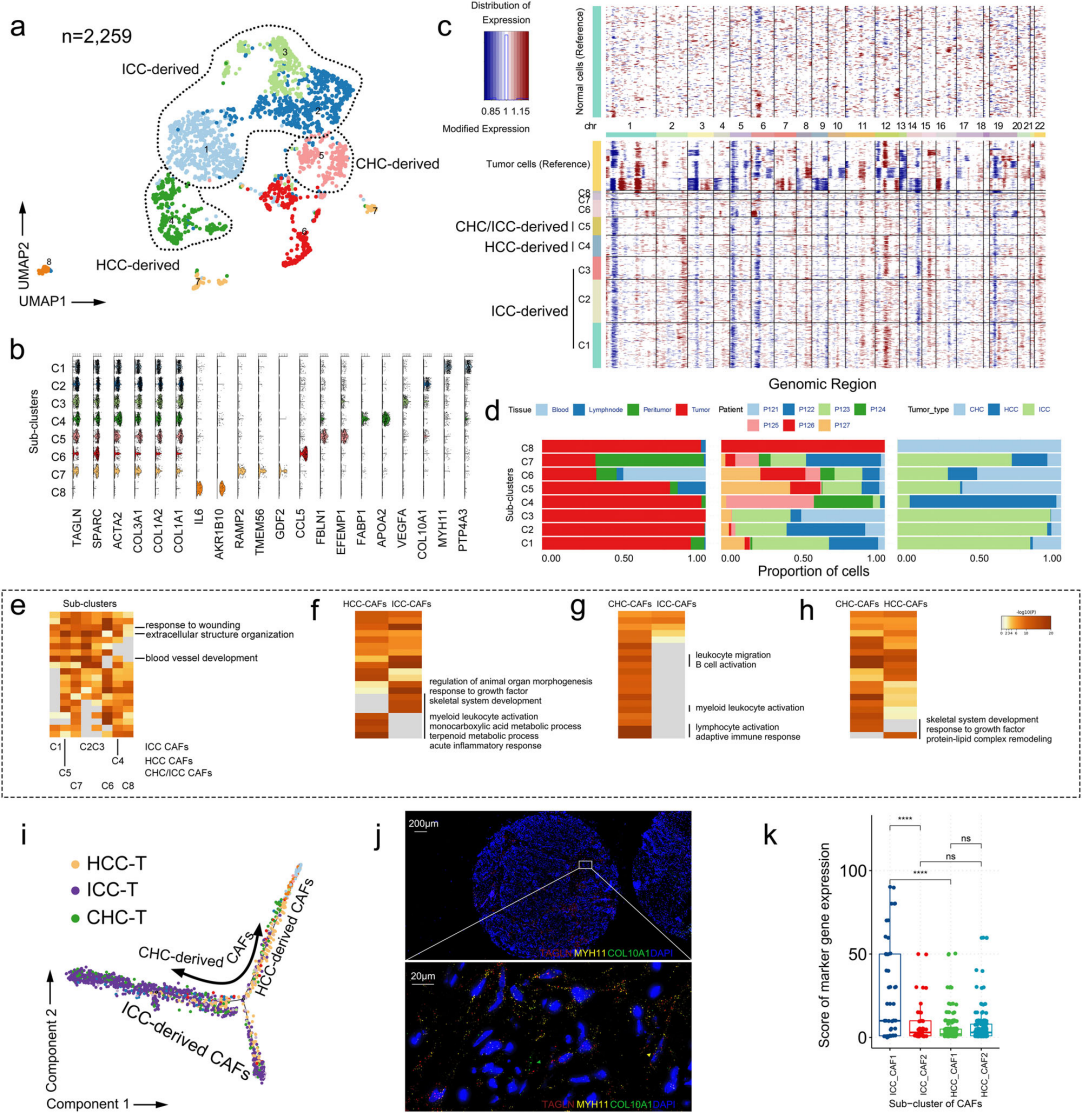
Identification of cancer-associated fibroblasts from PLCs. **a** UMAP distribution of re-clustered fibroblasts. ICC-derived, intrahepatic cholangiocarcinoma derived fibroblasts; HCC-derived, hepatocellular carcinoma derived fibroblasts; CHC-derived, combined hepatocellular-cholangiocarcinoma derived fibroblasts. **b** Stacked violin plot showing expression of canonical marker genes and differential expression of genes of sub-clusters. **c** Comparison of somatic chromosomal CNV between fibroblasts and tumor cells. **d** Bar plots of fibroblast sub-clusters based on tissue type, patient, tumor type. **e** Metascape pathway enrichment based on differentially expressed genes of each sub-cluster. **f** Differences of metascape pathway enrichment between HCC-CAFs and ICC-CAFs. CAFs, cancer-associated fibroblasts. **g** Differences of metascape pathway enrichment between CHC-CAFs and ICC-CAFs. **h** Differences of metascape pathway enrichment between CHC-CAFs and HCC-CAFs. **i** Pseudotime trajectory of CAFs derived from 3 PLC types. HCC-T, cells from HCC-derived tumor tissue; ICC-T, cells from ICC-derived tumor tissue; CHC-T, cells from CHC-derived tumor tissue. **j** RNAscope ISH stained with marker genes of subcluster C1-CAFs and C2-CAFs. Staining shows expression of C1 marker gene *MYH11*, C2 marker gene *CLO10A1*, and a common co-localization marker gene *TAGLN*. All nuclei were DAPI-stained. Scale bars, 200μm (upper), 20μm (lower). **k** Box plots presenting differential expression of MYH11 + C1 CAFs (left) and COL10A1^+^C2 CAFs (right) between HCC-T (*n* = 89 biologically independent samples, 2 replicates) and ICC-T (*n* = 48 biologically independent samples) external cohort. Two-sided t-test: ns, no significant; ****, *p* < 0.0001. Lower, inside and upper horizontal line of the box plot indicate first quartile, median, and third quartile, separately.

To investigate molecular signatures of fibroblasts in PLC, we re-clustered supra-cluster F (2259 cells), and obtained 8 sub-clusters (C1-C8). Sub-clusters displayed distinct characteristics of gene expression, and were identified with canonical marker genes (Fig. 4a, b). In our CNV analysis, cells of subclusters presented less CNV than heterogeneous malignant cells, suggesting that they were fibroblasts rather than malignant cells^44^ (Fig. 4c). Unlike malignant cells with heterogeneity at both tumoral and individual levels, fibroblasts from the same PLC type showed homogeneity over different patients (e. g. C1 was composed of cells from all three ICC patients), and these cells of sub-clusters were mainly obtained from tumor tissues, indicating that these fibroblasts were cancer-associated fibroblasts (CAFs) (Fig. 4d).

Consistent with prior understanding of CAF abundance in ICC^21^, the largest three sub-clusters were from ICC tumor tissues (C1, C2, C3, 60.29%), while only one sub-cluster (C4, 12.04%) was of HCC-specific derivation, and C5 was derived from CHC and its metastatic lymph nodes and ICC (Fig. 4d). Most sub-clusters resided in an active state and presented known functions, such as blood vessel development, extracellular matrix organization and response to wounding (Fig. 4e). Next, we explored the molecular characteristics of CAFs derived from different tumor types. CAFs from ICC exhibited enriched features of morphogenesis, skeleton development, and response to growth factor, while those from HCC presented enriched features of myeloid leukocyte activation and metabolic process, and those from CHC harbored both features mentioned above. Notably, ICC-derived CAFs appear to acquire more matrix remodeling functions, with a decreased level of immune activation (Fig. 4f-h). We also used the monocle 2 algorithm to establish a pseudo-temporal ordering of CAFs. ICC-derived and HCC-derived CAFs were located at opposite ends of the pseudotime trajectory, evidencing their differences in gene expression profiles. CHC-derived CAFs were located in the middle of the trajectory, validating that it involved dual features of ICC- and HCC-derived CAFs in gene expression profiles (Fig. 4i). Hence, we convincingly show that the functions of CAFs are driven by tumor types and their tumor microenvironment.

To explore the existence of CAF subtypes and the specificity of their marker genes, we constructed the tumor-tissue microarray of external cohort including 90 HCC (1 missing clinical information) and 48 ICC patients’ tumor tissues (1 tumor-tissue microarray defect) (Supplementary Table 2). We targeted *MYH11* (C1), *CLO10A1* (C2) and *TAGLN* (a common fibroblast co-localization marker gene) using RNAscope ISH, finding that *MYH11* and *COL10A1* were expressed separately, and that *TAGLN* was co-expressed (Fig. 4j). Statistically, ICC-derived MYH11^+^C1-CAFs significantly increased compared with HCC-derived counterpart, however, there was no statistical difference between ICC- and HCC-derived COL10A1^+^C2-CAFs (Fig. 4k). It pointed to the possibility of identifying CAF subtypes of ICC, considering the lack of fibroblast-specific markers^21,44^. Moreover, MNN and MIA analysis showed CAFs were more located in T and J zones (Supplementary Fig. 4a), and CibersortX analysis showed ICC bulk tumor samples had a higher proportion of fibroblasts compared with HCC counterpart, validating the SC data that ICC was the main CAFs’ source contributing to matrix remodeling and tumor progression (Supplementary Fig. 3g). Cell interaction analysis displayed CAFs had tight interaction with cells of myeloid and TNK supra-clusters (Supplementary Fig. 4b). *TIMP1-FGFR2* appeared to be an important pair between ICC-derived CAFs and these immune cells, which plays an essential role in the regulation of cell proliferation, differentiation, migration and apoptosis (Supplementary Fig. 4c), Of interesting, targeting FGFR2 was reported as a potent therapeutic potential for ICC with *FGFR2* fusions or rearrangements^45^. Overall, we extensively investigated single-cell molecular signatures of CAFs in PLC, and found them more prevalent in ICC than in other PLCs. We also found a relatively abundant MYH11^+^ CAFs spreading in ICC. All these hinted that CAFs-targeted therapeutic strategies might be more effective in ICC treatment.

### Endothelial cells in the tumor-peritumor junctional zone reside in an intermediate state

**Fig. 5:**
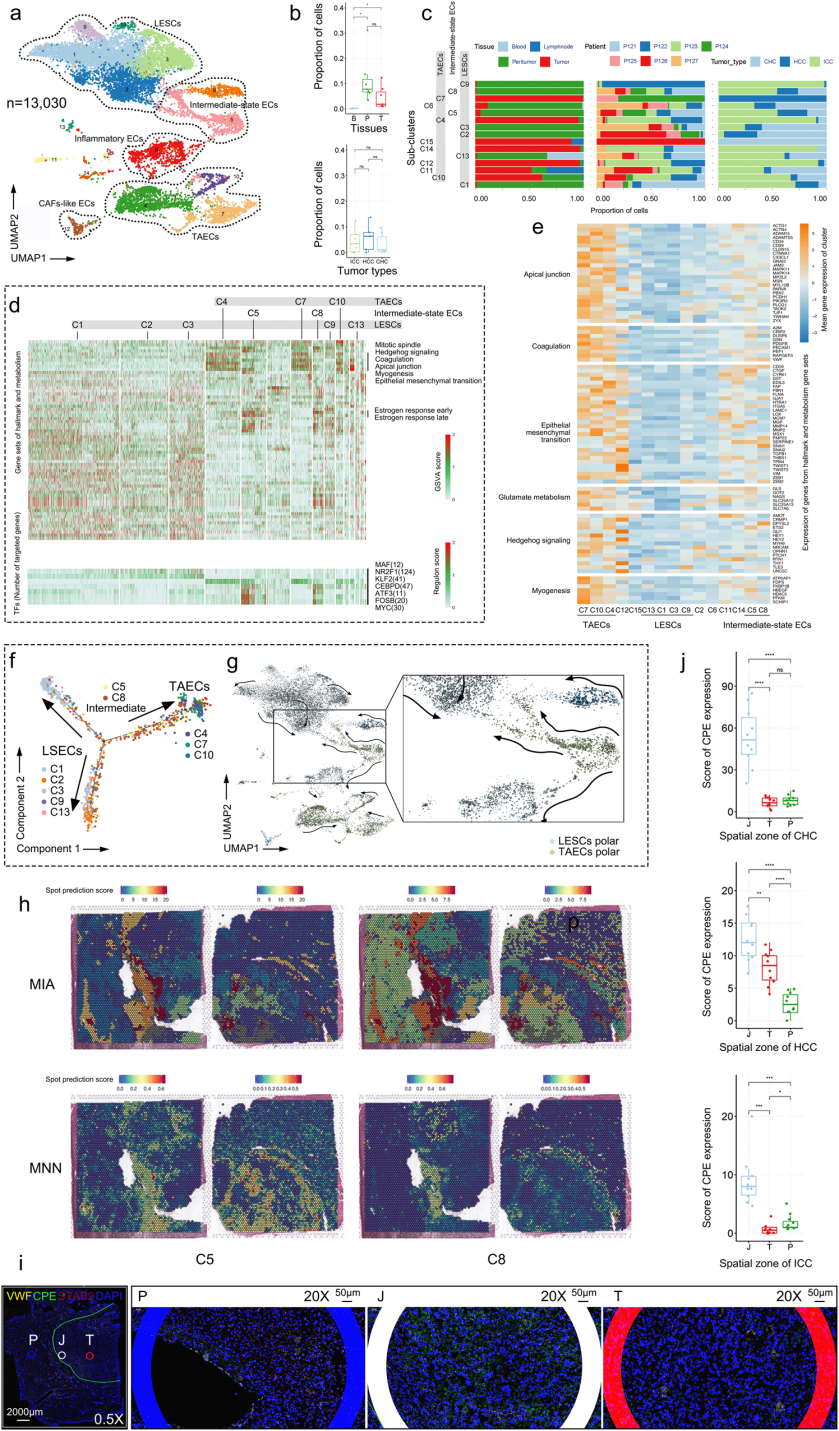
Identification of intermediate-state endothelial cells. **a** UMAP distribution of re-clustered endothelial cells. LSECs, liver sinusoidal endothelial cells; TAECs, tumor-associated endothelial cells; intermediate-state ECs, intermediate-state endothelial cells; inflammatory ECs, inflammatory endothelial cells; CAFs-like ECs, cancer-associated fibroblasts-like endothelial cells. **b** Box plots presenting the proportion of endothelial cells in terms of tissues (upper) and tumor types (lower). Two-sided t-test: ns, no significant; *, *p* < 0.05. **c** Bar plots of endothelial cell sub-clusters based on tissue, patient and tumor type. **d** GSVA of endothelial cell sub-clusters (upper) and SCENIC analysis of endothelial cell sub-clusters (lower). Light gray indicates lower enrichment and red indicates higher enrichment. **e** Expression heatmap of selected genes in endothelial cell sub-clusters. Blue indicates lower expression and yellow indicates higher expression. **f** Pseudotime trajectory of LSECs, TAECs and intermediate-state ECs. **g** RNA velocity showing dynamic flows among endothelial cells. Arrows show the directions. **h** Spatial distribution prediction of intermediate-state sub-clsuters C5 and C8 in P129 ST slices using MIA and MNN algorithm. Red color shows higher possibility of cells located in clusters of ST spots. **i** RNAscope ISH stained with marker genes of intermediate-state sub-cluster C5. Staining shows expression of C5 marker gene *CPE*, 2 common colocalization marker genes *VWF* and *STAB2*. All nuclei were DAPI-stained. T, tumor zone; P, peritumor zone; J, tumor-peritumor junctional zone. Scale bars, 2000μm (left), 50μm (right three). **j** Box plots presenting expression of CPE^+^C5 intermediate-state ECs based on different zones of slices over CHC (upper), HCC (middle) and ICC (lower). Two-sided t-test: ns, no significant; *, *p* < 0.05; **, *p* < 0.01; ***, *p* < 0.001; ****, *p* < 0.0001. Lower, inside and upper horizontal line of the box plot indicate first quartile, median, and third quartile, separately.

To decipher the atlas of endothelial cells of PLC, we re-clustered two EC-related supra-clusters (13,030 cells), and obtained 15 sub-clusters, including CLEC4G+ liver sinusoidal ECs (LSECs: C1, C2, C3, C9, and C13), tumor-associated ECs (TAECs: C4, C7, and C10), intermediate-state ECs (C5 and C8), and other ECs (Fig. 5a). As expected, ECs were most derived from peritumor and tumor tissues, and there was no statistical difference in EC proportion among tumor types (Fig. 5b). TAECs presented tumoral heterogeneity, among which C4 was mainly comprised of cells from ICC tumor tissues, and C7 from HCC tumor tissues. However, both LSECs and intermediate-state ECs did not display distinct heterogeneity at tumoral and individual levels (e.g., C1 was mainly composed of cells from all 7 patients’ peritumor tissues) (Fig. 5c). From the single-cell perspective, both TAECs and LSECs showed genomic stability and less copy number variation. Therefore, we considered LSECs as a negative reference for chromosomal alteration investigation (Supplementary Fig. 5a-c).

Next, we focused on TAECs, LSECs and intermediate-state ECs, employing GSVA, SCENIC, pseudotime trajectory and RNA velocity to analyze their differences in molecular signature. GSVA analysis showed that hallmark gene sets of EMT, myogenesis, apical junction, coagulation, hedgehog and mitotic spindle were enriched in TAECs (Fig. 5d). As described above, we found ECs and malignant cells were very likely to be co-localized in one ST spot (Supplementary Fig. 1e). Additionally, we found that ECs had tight communication with fibroblasts, M1, M2 and TNK through ligand-receptor interaction analysis (Supplementary Fig. 5d). *CD74-MIF* was also found to be the most pervading pairs between ECs and fibroblasts, which played a critical role in MHC class II antigen processing and inflammatory regulation of fibroblast proliferation (Supplementary Fig. 5e). These results suggest endothelial cells residing in tumor zone might closely interact with tumor cells or CAFs, acquiring more molecular alteration and participating in tumor ecosystem. The intermediate-state ECs significantly expressed genes enriched in early and late estrogen response, which may be associated with tumorigenesis^46^. However, LSECs did not exhibit distinct gene set enrichment of hallmark or metabolism (Fig. 5d). We further filtered genes from hallmark gene sets associated with TAECs, observing an increase in gene expression profiles from LSECs to intermediate-state ECs to TAECs (Fig. 5e). SCENIC analysis identified *MAF* and *NR2F1* as the key TFs of LSECs in cell differentiation, especially for T-helper-2 (Th2) cell, and *KLF2* was identified as the key TF of TAECs in promoting T-cell quiescence^47^. These results indicate the phenotype differences between LSECs and TAECs. Of interest, intermediate-state ECs, in which *CEBPD*, *ATF3*, *FOSB*, and *MYC* were identified as the underlying TFs functioning cell cycle regulation and self-renewal properties, is functionally similar to the recently reported fetal-like endothelial cells^25^ (Fig. 5d). In the pseudotime trajectory, LSECs and TAECs were located at opposite ends of the trajectory, with intermediate-state ECs in the middle (Fig. 5f). In addition, RNA velocity depicted that intermediate-state ECs exhibited two directional flows toward LSECs and TAECs (Fig. 5g). All the evidence implies that intermediate-state ECs involved two-edged potential of functionality from LESCs and TAECs.

We further investigated whether the functionality of intermediate-state ECs might connect to their spatial distribution pattern. Through MNN and MIA algorithm, we inferred that intermediate-state ECs were located in J zones of P129TP1 (C10) and P129TP2 (C9) (Fig. 5h). To verify that, we selected 3 SC-matched tumor-peritumor tissue slices (covering 3 tumor types), and detected intermediate-state sub-cluster C5 marker (*CPE*) and two EC co-localization markers (*VWF* and *STAB2*) using RNAscope ISH. We found that *CPE* expression was spatially consistent with the expression of *VWF* and *STAB2*, and *CPE* was more highly expressed in J zone than in T or P zone (Fig. 5i, j, Supplementary Fig. 5f), which accorded with our inference. The spatial distribution of intermediate-state ECs (more located in the region between tumor and peritumor) were in line with their intermediate state of functionality. Together, we identified a population of intermediate-state cells that display an intermediate state of functionality between LESCs and TAECs, which might relate to their spatial distribution in J zone.

## Discussion

Characterizing stochastic heterogeneity within tumor microenvironment as a persisting challenge is critical to understanding how tumors develop and their molecular mechanisms. Given advances in single-cell technologies, scRNA-seq has provided an unbiased approach for profiling cell diversity and lineage trajectories in many different tumor types^26,48,49^. There were certain previous scRNA-seq attempts to construct single-cell landscapes of HCC or ICC, in which challenges remained regarding the extensiveness of PLC investigation^6,21–23,25,38^. Of this, scRNA-seq and spatial technologies provided many layers of information to defining PLC types including CHC. Here, using single-cell transcriptomics, spatial transcriptomics, and bulk multi-omics, we constructed a comprehensive molecular architecture of PLC, comprising ICC, HCC and CHC, featuring tumor microenvironment and spatial heterogeneity. We surveyed ~289,156 cells to conduct a comparative analysis among ICC, HCC and CHC microenvironment, involving cancer cells, immune cells, cancer-associated fibroblasts and endothelial cells. We revealed an intermediate-state endothelial cell subset with distinct phenotype, and investigated its spatial distribution pattern. These results offer insights into homogeneous and heterogeneous characteristics of PLC microenvironment, contributing to broad implications for drug development and pathological investigations.

Malignant cells from all 3 PLC types manifested the highest level of individual heterogeneity (compared with non-parenchymal cells). This might explain the limitations of cytotoxic drugs that were indiscriminately targeted at cancers^50^. Using ST combined with scRNA-seq data, we stereoscopically observed the phenotype differences between HCC and ICC, with HCC manifesting abnormal metabolic enrichment in fatty acid metabolism, bile acid biosynthesis, cholesterol homeostasis, and abnormal metabolism of monocarboxylic acid, and ICC manifesting myogenesis and EMT. It suggests explorations into more precise therapeutical strategies which target metabolic abnormalities at HCC, and EMT at ICC^50–52^. CHC exhibited internally discordant phenotypes which combined with characteristics from HCC and ICC malignant cells, while presenting a specific transcriptional regulation that was different from HCC and ICC. Its further exploration of therapeutic strategies is more challenging. The phenotypes of CAFs were consistent with those of their corresponding PLC types. ICC-derived CAFs exhibited enriched features of morphogenesis, skeleton development, and response to growth factor, while HCC-derived CAFs presented enriched features of myeloid leukocyte activation and metabolic process, and CHC-derived CAFs harbored both features mentioned above. This suggests CAFs play a crucial role in constructing PLC phenotypes. Additionally, based on scRNA-seq, CAFs were also found more abundant in ICC than in HCC and CHC from a single-cell perspective. Among these, CAFs derived from ICC were categorized into three distinct sub-clusters based on their fibroblast-restricted markers. Notably, MYH11^+^CAFs were found to be more prevalent in ICC tumor tissues compared to HCC tumor tissues. Targeting strategies aimed specifically at these sub-clusters could potentially impact the EMT process, thereby facilitating the accomplishment of precise anti-ICC therapeutic objectives^21,44^. However, the precise functional roles of ICC-derived CAFs remain largely unknown and require future investigations^53,54^. Difference in phenotypes has also been validated with bulk multi-omics. Metabolic process of monocarboxylic acid was discovered in bulk HCC samples with ATAC-seq, mRNA-seq, lncRNA-seq and proteomics. Extracellular structure organization was found a significant enrichment in ICC and CHC bulk tissues, and miRNA appeared to be a critical regulator of the process^55,56^. By in-depth exploration of miRNA that plays an important role in EMT regulation, it provides a feasible path to precise ICC therapeutical strategies through targeted regulation of the process of EMT.

Tumor-peritumor junctional zone is rarely elaborated with high-throughput technology. In this study, J zone exhibited a much more complex gene expression pattern compared with T or P zone which displayed a relatively consistent phenotype respectively. The function of cells near J zone were affected by tumor microenvironment, such as the intermediate-state cells. We found and verified the existence of subcluster of intermediate-state ECs (C5 marked with *CPE*) residing in J zone, which involves the potential of functionality from LESCs and TAECs. The sub-cluster was functionally similar to the recently reported fetal-like endothelial cells in HCC^25^. We also found diverse intermediate-state CD8^+^ T cells pervading in PLCs, functionally consistent with previously described GZMK^+^ CD8^+^ T cells in HCC^22^. These cells were more likely to be located in J and S zones, suggesting their intermediate-state function might link to their spatial distribution. These intermediate-state cells might play an essential role in cell transition from naïve to effector to exhausted T cells, serving as a target or predictive marker for immunotherapy and more. In practice, we observed increased effector and decreased naïve immune cells after tumor burden removal with curative hepatectomy (Supplementary Note 1. Supplementary Figs. 6 and 7). Overall, the analysis revealed a diverse population of cells residing in the J zone, suggesting that this zone could potentially serve as a targeted region for precise therapeutic strategies. By modulating the functionality and abundance of intermediate-state cell subsets within the J zone, it may be possible to develop targeted interventions. We look forward to further investigations focused specifically on the J zone to gain a deeper understanding of its potential in therapeutic applications.

Due to unsupervised cell collection strategy, we noticed immune cells did not display statistical difference in cell composition among different PLCs, which validates the efficacy of immunotherapy for a wide range of malignant tumors, such as melanoma, non-small cell lung carcinomas (NSCLC) and colorectal cancer (CRC) et al. ^50–52,57–59^. We observed that HCC gain much more patient-specific heterogenous T cell clusters, that ICC acquired relatively abundant clonal diversity of TCRs repertoire, and that CHC appeared to be of a moderate performance. These results, in part, could offer a clue about personalized immunotherapy for different tumor types. Besides, we observed two clusters of highly proliferative cells, TXNDC5 + B cells and TYMS+ myeloid cells. These cells might play a critical role in tumor invasion and metastasis, which is worth further investigation.

In terms of the cell dissociation process of tissues for scRNA-seq, certain minor cell types might have faced the risk of being lost. Unsupervised cell collection and a series of deconvoluting algorithm including MNN, MIA and RCTD has been employed to minimize the risk, and to better connect modalities between SC and ST. The inevitable tumor heterogeneity might affect the results and generate possible batch effects. In practice, we collected matched peritumor tissues for reference control and did not observe batch effects in the results. The limited number of samples analyzed in our study is a potential limitation that could affect the generalizability of our findings. Although we analyzed multiple modalities, including bulk RNA sequencing, single-cell RNA sequencing, and spatial transcriptomics, we only analyzed a small number of samples, particularly for the spatial analysis. As a result, it is difficult to account for inter-patient variability, such as variations in age, gender, cancer stage, and treatment history, which could affect the observed molecular phenotypes.

Despite this limitation, our study provides a comprehensive landscape of PLC featuring 3 main tumor types with multi-dimensional high-throughput approaches. Our findings highlight the heterogeneity of these cancers and the importance of considering spatial context and microenvironment in understanding tumor biology. We hope that our study will inspire further research in this field, and contribute to the development of diagnostic and therapeutic strategies for these deadly cancers.

## Methods

### Ethical statement, human tissue collection, dissociation and preparation of single-cell suspensions

The research was approved by Ethics Committee of Zhongshan Hospital, Fudan University (approval number B2019-216R). Informed consent was obtained from each patient for the collection and research of surgically removed liver and peripheral blood samples. All employed protocols in this study abided by the ‘Regulations on the Management of Human Genetic Resources’ administered by The Ministry of Science and Technology (approval number 2021BAT0574, 2022BAT1853). Details on donor information are provided in Supplementary Table 1. All included patients did not receive any pre-operative adjuvant therapy, and their diagnoses were pathologically confirmed by at least two pathologists. The paired fresh solid tissues were obtained immediately after resection with tissue storage solution (miltenyi biotec, 130-100-008), minced on ice to smaller pieces less than 2-4 mm^3^, transferred into 10 ml 1 mg/ml dulbecco’s modified eagle medium (DMEM, GIBCO) containing 1x collagenase type I (GIBCO, 17100-017) and type IV (GIBCO, 17104-019), and incubated at 220 rpm 37°C oscillator for 10-25 min depending on tissue hardenability (details provided in Table S1). Samples were then vortexed for 1 min using the gentleMACS™ dissociator at spleen IV mode and filtered using 40-μm nylon mesh (FALCON ThermoFisher). Following centrifugation at 200 g at 4°C for 5 min, the supernatant was discarded, and the cell pellet was resuspended in 2 ml 1x red blood cell lysis buffer (BD Biosciences, 00-4333-57), transferred to a new 5 ml centrifuge tube following incubation on the ice for 15 min and centrifuged (200 g, 4°C, 5 min). Samples were next resuspended in 1 ml stain buffer (BD Biosciences, 554656) and washed (200 g, 4°C, 5 min) twice. To prepare for blood samples, 400 μl blood was mixed with 2 ml red blood cell lysis buffer, incubated on ice for 15 min, and centrifuged at 200 g at 4°C for 5 min. The supernatant was discarded, and the process was repeated once. Single-cell suspension was finally resuspended in 1 ml stain buffer and washed (200 g, 4°C, 5 min) twice. Suspensions were tested and stained with 4% trypan blue (1 suspension: 9 trypan blue), and cell viability was calculated under microscope with cell counting plate. Cells with viability >80% were enrolled for further processing. Dead Cell Removal Kit (MACS, 130-090-101) were employed in cells with unsatisfactory viability (Supplementary Data 1b). Tissues and cells were maintained on ice whenever possible. In this study, we define the area 1 cm inside and outside the edge of tumor as the junctional zone; the area outside the edge of tumor as the peritumor zone; the area inside the edge of tumor as the tumor zone. It is noted that the junctional zone overlaps the other two zones, and tissues of peritumor zone was obtained from the area that is more than 1 cm away from the edge of tumor.

### Single-cell library preparation

10X Chromium 5’ gene expression single-cell reagent v1.0 kit (10X Genomics) was used for single-cell and V(D)J library preparation immediately after the samples satisfied the manufacturer’s protocol. Agilent 2100 bioanalyzer with high sensitivity DNA chip (Agilent Technologies) was employed for quality control of cDNA and final libraries. Sequencing was performed on a Nova-seq 6000 (Illumina) at a median depth of 61,589 reads/cell (Supplementary Data 1a).

### Spatial slide preparation, permeabilization optimization, imaging and spatial gene expression library construction

PLC tissue (1–2 cm^3^) was gently washed with cold PBS and frozen in OCT-filled mold. RNA quality was examined by Agilent 2100 bioanalyzer and satisfied with RNA Integrity Number (RIN) ≥ 7. The frozen tissue was trimmed into 6.5 mm^3^ pieces, and the cryosections (10 µm thickness, 7 of the 8 Capture Areas, 1 empty for positive RNA control) were mounted onto the Visium Spatial Tissue Optimization Slide (10x Genomics). The slide was incubated at 37°C for 1 min and immersed in the pre-chilled methanol at -20°C for 30 min. Next, 500 μl isopropanol uniformly covered the tissue sections to dehydrate at room temperature for 1 min, and the sections were stained with H&E. Slides were mounted in 80% glycerol and bright-field images were taken (Leica SCN400 F whole-slide scanner, 40× resolution). Tissue optimization slide was incubated with permeabilization enzyme for different time settings, fluorescent cDNA synthesis was performed at 53 °C for 45 min, and the tissue was removed following manufacturer’s instructions. Fluorescent images were taken, and optimum permeabilization time was identified with maximum fluorescence signal for spatial gene expression. The cryosection was remounted to The Visium Spatial Gene Expression Slide (10x Genomics), which included 4 capture areas with 5000 gene expression spots in each area [primer, including Illumina TruSeq Read 1, 16 nt Spatial Barcode, 12 nt unique molecular identifier, 30 nt poly(dT) sequence]. Tissue fixation, H&E staining, bright-field imaging, permeabilization and reverse transcription were performed as previously described and following manufacturer’s instructions. Second strand mix was added to the tissue section for second strand synthesis (65 °C for 15 min), followed by denaturation and transfer of cDNA from capture area to a corresponding tube for amplification and library construction (98 °C for 3 min, followed by 15-16 cycles at 98 °C for 15 s and at 63 °C for 20 s). Sample quality control and quantification were assessed by Agilent 2100 bioanalyzer and Agilent 2100 Expert Software. Purification of cDNA sample (10 µl), paired-end, and dual indexed sequencing were performed on Nova-seq 6000 with 150 cycles for read 1, 10 cycles for i7 index, 10 cycles for i5 index and 150 cycles for read 2.

### Spatial validation using multiplex fluorescent in-situ RNA hybridization (RNAscope ISH) and fluorescent multiplex immunohistochemistry (mIHC)

Formalin-fixed paraffin-embedded (FFPE) fresh tissue blocks were trimmed and cut into 5 + /-1 µm sections using a microtome. Slides were stained using the RNAscope Multiplex Fluorescent Reagent Kit v2 Assay and RNAscope 4-plex Ancillary Kit (ACDBio) manually according to the manufacturer’s protocol. RNAscope target probes were run with parallel of multiplex positive and negative controls (cat#. 321831, 321801). All nuclei were DAPI-stained. All images were scanned with Pannoramic MIDI (3D HISTECH) and FV3000 (OLYMPUS). Opal fluorophore working solution and channels were Opal 520 (1:750, Excitation 488 nm, Emission 500-540 nm, FITC), Opal 570 (1:750, Excitation 561 nm, Emission 570-620 nm, Cy3), Opal 690 (1:750, Excitation 640 nm, Emission 650-750 nm, Cy5), and DAPI (Excitation 405 nm, Emission 430-470 nm). Images were viewed and processed with Caseviewer (version: C.V 2.3). All antibodies used for mIHC are commercially available and their manufacturers provided their validation documents: SMA (1:200), Cat# BM0002, boster; Vimentin(1:1000), Cat# 10366-1-AP, PTG; Fibronectin(1:500), Cat# 66042-1-Ig, PTG.

### Single-cell RNA-Seq and ST data processing

Analysis pipelines (Cellranger 3.0.1) for single-cell RNA-seq and space Ranger (version 1.0.0) for spatial RNA-seq output were employed to generate feature-barcode and feature-spot matrices respectively, which were mapped to the hg38 reference genome. Seurat R package (version 3.2.0) was then employed and threshold values (single cell with ≥ 200 genes was detected, with < 10% red cell gene mapped reads, and with < 10% mitochondrial gene mapped reads) were included into downstream analyses. We employed *scrublet* to remove doublets, and there is no clear positive correlation between cell capture and doublets (Supplementary Data 1b). All filtered cells were integrated, corrected for batch effect with SeuratV3, and normalized to total cellular read count. Highly variably expressed genes were selected with FindVariableFeatures function and summarized by principle component analysis (proportion of mitochondrial read count as a variable for regression). For dimensionality reduction, RunUMAP Seurat function (Seurat version 3.2.0) and Louvain algorithm were performed using the robust settings (informative principle components=50, k-means =30, Resolution=0.6), and the results of running with varying parameter settings were evaluated with normalized mutual information (NMI). To assess the effects of cell proliferation, cell cycle regression was employed to compute cell cycle phase of each cell based on canonical markers, and they were regressed out using ScaleData function. Clustering was re-executed and compared with pre-regression clusters using NMI. Spatial spots were processed like single-cell barcode (informative principle components=50, k-means =30, Resolution=0.9).

### Cell type annotation

To determine cell types, we filtered differentially expressed genes between target cluster and other cells using FindAllMarkers function in Seurat (*P* value ≤ 0.05, log2FC ≥ 0.585). The cells were annotated with cell-type-specific expression known in the literature. Moreover, SingleR and SCINA, two built-in reference annotation methods, were employed to verify the cell-type assignment. The broadly defined categories (supra clusters) were further assembled on the basis of same biological cell types such as myeloid, endothelial, etc. Repeating dimensionality reduction and unsupervised clustering were performed with those cell types, and they were re-clustered into more specific cell subsets with similar modules. When defining a cluster specified by a marker gene, the average expression <1 in other subclusters was required and the marker gene was selected in consideration of its biology function. Mixed cell sub-cluster (or supra-cluster) was defined as a cluster that includes two or more canonical marker genes of different cell types in the differentially expressed gene list, and presents an undistinguished characteristic. Reference of the Human Primary Cell Atlas (Mabbott et al. 2013) and Blueprint (Martens and Stunnenberg 2013) and Encode (The ENCODE Project Consortium 2012) were employed.

### Gene set variation analysis (GSVA), Metascape, SCENIC and velocity analysis

Pathways were enriched on the hallmark and metabolic gene sets reported previously, and GSVA package (version 1.36.2) were used with standard vignette. For enrichment analysis of significant genes in each cluster or group, we employed Metascape, an automatical gene enrichment visualization tool, to assign the biological function of the clusters. To expand the interaction between transcription factors (TFs) and target genes using scRNA-seq data, we utilized single-cell regulatory network inference and clustering (SCENIC) to score the activity of each regulon in each cell (pyscenic, version: 0.10.3, https://github.com/aertslab/pySCENIC). Velocyto was employed to produce loom for RNA velocity of single-cell RNA-seq data (velocyto: 0.17.17, https://github.com/velocyto-team/velocyto.py) and scvelo for pseudotime (scvelo: 0.2.2, https://github.com/theislab/scvelo).

### Trajectory analysis and diffusion component analysis

Monocle (version 2) algorithm was employed to calculate the potential lineage differentiation of cell groups. For computational efficiency, the samples were downsized to 2000 for those groups over 10000 cells. CellDataSet object was created with the parameter “expressionFamily = negbinomial.size” and positioned along the trajectory based on the differentially expressed genes in clusters. After dimension reduction and cell ordering with the default parameters, the cell was visualized and inferred with tissue, tumor type, state, pseudotime, etc. Density diffusion Map (Version: 3.2.0) was applied in this study^60^.

### CNV analysis of SC data

To establish CNV profiles, we first annotated scRNA-seq genes of their chromosomal location using AnnoProbe R package. For computational efficiency, the samples were down-sized to 5000 cells for those groups over 10000. We then used CreateInfercnvObject function in infercnv R package to run the standard vignette. The cutoff 0.1 was set as recommended. For negative control, we sampled endothelial cells from peritumor tissues as the reference.

### T-cell receptor (TCR) and B-cell receptor (BCR) repertoires analysis

Clonotype was defined as a group of T/B cell clones with equal amino acid CDR3 sequence and V gene combined alpha and beta chains for TCR (heavy and light chains for immunoglobulin). R package^61^ tcR was used for repertoire statistic, quantification assessment and diversity evaluation including inverse Simpson and ecological diversity index. Epitope was annotated with VDJ database (https://vdjdb.cdr3.net/).

### Cell interaction analysis

To profile and visualize intercellular communication between clusters, ligand-receptor database CellPhoneDB (https://www.cellphonedb.org/downloads; http://fantom.gsc.riken.jp/5/suppl/Ramilowski_et_al_2015/data/PairsLigRec.txt) were applied. The preliminary characterization was performed for the eight supra clusters, which was filtered with more than 20 paired ligand-receptors and further processed in the identified sub-clusters.

### Integration SC and ST data

MIA was employed to calculate the overlap between single-cell cluster and ST cluster based on marker genes using the hypergeometric cumulative distribution. We employed an ‘anchor’-based integration (Seurat v3) to predict each ST spots’ probabilistic classification derived from scRNA-seq labeled clusters (MNN). Robust cell type decomposition (RCTD) was employed to decompose cell types of ST spots learned from SC data.

### Combination analysis of bulk RNA-seq and scRNA-seq

CibersortX (https://cibersortx.stanford.edu/) were used to estimate and predict gene expression profiles to the abundances of member cell types. Of note, 2000 random single-cell profiles from eight supra clusters were imputed to CibersortX as a reference signature.

### Bulk sample extraction, QC, library preparation, sequencing or LC-MS/MS

#### Nuclei extraction and Tn5 transposome based assay for transposase-accessible chromatin

Frozen tissue (20 mg) for each sample was grinded and a total of 50,000 cell nucleis were used per reaction in the preparation of ATAC. Library construction was conducted with TruePrep DNA Library Prep Kit V2 (Vazyme Biotech) for Illumina according to the manufacturer’s protocol. Genomic DNA was fragmented with a hyperactive Tn5 transposase (Tn5 kit), which fragmentes DNA and appended sequencing adaptors in a single step. DNA library was purified using Minelute PCR Purification Kit (Qiagen) and amplified using TruePrep Index Kit V2 for Illumina (Vazyme Biotech). The cells were washed with cold PBS once. The supernatant was removed by 500 g centrifugation at 4°C for 5 min.

### Genomic DNA preparation for WES and RRBS

Genomic DNA was extracted from liver tissues using a genomic DNA extraction kit (TIANGEN, YDP341-T4A) according to the manufacturer’s protocol. DNA concentration, DNA degradation and contamination were analyzed by agarose gel electrophoresis. DNA quantity and purity were assessed using a Nanodrop (OD 260/280 ratio). For RRBS, genomic DNA digested with methylation-insensitive restriction enzyme (MspI) was size-selected and spiked-in with 0.5% fully methylated lambda DNA. DNA library was then prepared with EZ DNA Methylation Gold Kit (Zymo Research) according to the manufacturer’s protocol. DNA concentration and quantification were assessed by qPCR, and the fragment size was accessed by Agilent 2100 Bioanalyzer. WES libraries were prepared and captured using Roche NimbleGen SeqCap EZ Exome V3 and Agilent SureSelect Human All Exon V6 (Agilent Technologies) following the manufacturer’s instructions.

### Total RNA preparation for whole RNA-seq

Total RNA was extracted and purified from fresh tissue specimens using TRIzol (Thermo Fisher) according to the user guideline. Quality inspection of the extracted RNA samples were conducted by Agilent 2100 Bioanalyzer (Agilent Technologies). The qualified samples which had a total amount > 500 ng and RIN > 7 were included in the following library construction and sequencing experiment: 1. LncRNA library construction was performed according to the operation manual provided with KAPA RNA HyperPrep kit with RiboErase (HMR). 500 ng of total RNA depleted rRNA and treated with DNase was interrupted at an average size of 300-400 nt. The first and second cDNA strands were then biosynthesized from the fragments following the end-repair, A-base addition and sequencing adaptor ligation. 2. sRNA library construction was conducted according to the manufacturer’s protocol with NEBnext multiplex small RNA library prep kit (NEB). Qualified samples’ 3′ and 5′ adapters were ligated followed by reverse transcription reaction and cDNA synthesis. PCR amplicons were subsequently performed using PCR amplification primers in the ligation. Following PCR amplification, the DNA fragments of 140-160 bp were separated by PAGE. Agilent 2100 Bioanalyzer was used for quality inspection. The libraries were sequenced on Nova-seq 6000.

### TMT-labeled LC-MS/MS for proteomics

Tissue was grinded into cell powder with liquid nitrogen and then added four volumes of lysis buffer [8 M urea (sigma), 1% Protease Inhibitor Cocktail), followed by sonication three times on ice using a high-intensity ultrasonic processor (Scientz). The supernatant was collected after centrifugation (12,000 g at 4 °C for 10 min) and protein concentration detected with BCA kit (Thermo Fisher) according to the manufacturer’s instructions. The protein solution was reduced with 5 mM dithiothreitol (30 min at 56 °C), alkylated with 11 mM iodoacetamide (15 min at room temperature in darkness), and diluted urea concentration < 2 M by adding 100 mM triethyl ammonium bicarbonate (TEAB, sigma). The first digestion of proteins was added with trypsin at a ratio of 1:50 trypsin-to-protein overnight at 37°C and followed by second 4-h digestion with trypsin (1:100) at 37°C. Internal reference “MIX” sample was prepared with mixed samples in remaining channels and mixed in equal protein amount, which was also processed and conducted as a sample in TMT-labeling experiment. For each sample, 400 mg were desalted by Strata X C18 SPE column (Phenomenex) and vacuum-dried according to the TMT kit instructions (Thermo Fisher). Each TMT 11-plex reagent set was distributed ahead. Peptides were separated with a gradient of 8% to 32% acetonitrile (pH 9.0) over 60 min into 60 fractions by high pH reverse-phase HPLC (Agilent 300Extend C18 column, 5 μm particles, 4.6 mm ID, 250 mm length), then combined into 14 fractions, and dried by vacuum centrifugation for nanoscale liquid chromatography coupled with tandem mass spectrometry (LC-MS) using a 1290 Infinity series UHPLC System (Agilent Technologies). The fractionated peptides (1 mg each fraction) were dissolved in 0.1% formic acid (solvent A) and loaded onto a home-made reversed-phase analytical column (15-cm length, 75 μm i.d.) at a constant flow rate of 450 nL/min on an EASY-nLC 1200 UPLC system (Thermo Fisher Scientific). The gradient was set as follows: 9% -30% solvent B (0.1% formic acid in 80% acetonitrile) in 40 min, 30%-40% in 12 min, 40%-90% in 4 min and holding at 90% for the last 4 min. The peptides were then subjected to NSI source and performed tandem mass spectrometry (MS/MS) with Orbitrap Fusion Lumos (Thermo Fisher). The electrospray voltage was set at 2.4 kV and 400 -1500 m/z scan range for full scan at a resolution of 60,000, and then selected for MS/MS at 100 m/z scan range (resolution 30,000). Automatic gain control (AGC) target was set at 5E4, maximum IT at 100 ms, signal threshold at 10000 ions/s with 30 s dynamic exclusion time between MS1 and MS2 scan. A data-dependent procedure was applied, and the normalized collision energy (NCE) was set at NCE 32%. All MS/MS data were processed using Maxquant search engine (v.1.5.2.8) against the human Swiss-Prot database containing 20,380 sequences (downloaded in December, 2017) concatenated with reverse decoy database. Enzyme specificity was set as Trypsin/P allowing up to 2 missing cleavages. The mass tolerance for precursor ions in the first and main search was set as 20 ppm and 5 ppm, respectively, and the mass tolerance for fragment ions as 0.02 Da. The minimal peptide length was set at 7 and the maximum modifications per peptide at 5. The identified peptide length distribution most ranged in 7-20 amino acids which were eligible for quality control. Fixed modification (carbamidomethyl on Cys) and variable modifications (acetylation modification and oxidation on Met) were specified with FDR < 0.01.

### Metabolites Extraction

Sample tissues (25 mg) were dissolved in 500 μL extract solution (acetonitrile: methanol: water = 2: 2: 1) (acetonitrile 75-05-8, Methanol 67-56-1, CNW Technologies) and vortexed for 30 s, followed by homogenization at 40 Hz (4 min) and sonication (10 min) in ice-water bath for 3 times. Then the samples were incubated at -40 °C (1 h) and 450 μL of supernatant was transferred after centrifugation (12000 rpm, 15 min at 4 °C) and dried in 37 °C vacuum concentrator. The dried samples were reconstituted in 200 μL of 50% acetonitrile and sonicated in ice-water bath for 10 min. Supernatant (75 μL) was collected after centrifugation (13000 rpm, 15 min at 4 °C) for LC-MS/MS analysis using 1290 Infinity series UHPLC System (Agilent Technologies) and Triple TOF 6600 (AB Sciex). Besides, a mixture with above 10 μL supernatant from each sample were prepared for quality control. Fractionation was processed with UPLC BEH Amide column (2.1 * 100 mm, 1.7 μm, Waters). Solvent A consisted of 25 mmol/L ammonium acetate and 25 mmol/L ammonia hydroxide in water (pH = 9.75) and solvent B acetonitrile. The gradient was set as follows: 95% B (0–0.5 min), 95%-65% B (0.5–7.0 min), 65%-40% B (7.0–8.0 min), 40% B (8.0–9.0 min), 40%-95% B (9.0–9.1 min), 95% B (9.1–12.0 min). The parameters were set as fellows: 25°C column temperature, 4 °C auto-sampler temperature, 0.5 mL/min Flow rate of mobile phase and 1 μL injection volume (pos or neg). MS/MS spectra were acquired on an information-dependent basis (IDA) and collected with acquisition software (Analyst TF 1.7, AB Sciex) continuously depending on preselected criteria. The most intensive 12 precursor ions with intensity > 100 were chosen for MS/MS at 30 eV collision energy (CE) in each cycle (0.56 s). ESI source conditions were: 60 psi GS1, 30 psi GS2, 35 psi curtain GS, 600 °C source temperature, 60 V declustering potential, 5000 V (Pos)/-4000(Neg) ion spray voltage floating (ISVF).

### Bulk data processing and analysis

#### ATAC-seq

Cutadapt software were employed to remove adapters of sequenced reads and filtered short reads (<35 bp) accompanied with quality control (Reads with N ratio > 10% and bases quality value Q ≤ 10 occupied > 50% of the entire Read were removed). High-quality clean reads of each sample were mapped to human reference genome (ftp://ftp.ensembl.org/pub/release-95/fasta/homo_sapiens/dna/) using Bowtie2 software. MACS2 v2.2.7.1. was employed for peak calling and the empirical false detection rate (FDR) < 0.05 was selected as the identified peak. Genome-wide peak was analyzed by ChIPseeker package and annotated to the functional elements of each gene on the genome (TSS, 5 ‘UTR, 3’ UTR, Exon, Intronic or Intergenic region). The peak distribution was presented as heatmaps according to the peak-elements distance. MEME-ChIP 4.11.2 was employed to identify and annotate Motif, and Tomtom software to align the detected Motif sequence with known Motif (JASPAR database, http://jaspar.genereg.net/), which made it accessible to acquire transcription factor information. For Difference Peak analysis, DiffBind package was used to calculate the affinity score based on the number of standardized read count, which was then inputted to screen the differentially accessible region (DAR) using DESeq2 software (fold change > 1.5, *P* value < 0.05) giving to subscribed group. DAR, referred to promoter region <1 kb, was highlighted in our analysis. Enrichment analysis was performed with Metascape.

### RRBS

The clean reads from RRBS were mapped to human reference genome GRCh38 (ftp://ftp.ensembl.org/pub/release-95/fasta/homo_sapiens/) using bismark software, which also processed 5mC detection and annotation (coverage > =4X and FDR < 0.05). Different methylation region (DMR) was calculated with MOABS and qualified with coverage > =4X and different methylation sites >=3 and methylation variation >=0.2 (fisher’s exact test, *p* < 0.05).

### Whole exome sequencing

Raw data were filtered to clean data as ATAC-seq described and mapped to the human reference genome (http://grch37.ensembl.org/Homo_sapiens/Info/Index) using BWA mem (version 0.7.12). The Picard tool (https://github.com/broadinstitute/picard) was used for duplication sortation and marker. Single nucleotide variants (SNVs) and short insertions and deletions (INDELs) were analyzed with mutect2 package. Control-FREEC was employed for somatic CNV and B Allele Frequency (BAF). Maftools R package was used to analyze mutational signatures and disassemble into distinct signatures based on non-negative Matrix Factorization (NMF) algorithm, which was then mapped to the COSMIC database (http://cancer.sanger.ac.uk/cosmic/signatures) for mutational signature decoding. Gain-of-function mutation was predicted with OncodriveCLUST or screened from the Cancer Gene Census (CGC) database or published paper. Predisposing gene was inquired from Germline Mutation (SNV, InDel) using GATK software (HaplotypeCaller) and searched in CGC database. Driver genes were filtered with the known driver mutations (http://cancer.sanger.ac.uk/cancergenome/projects/census/cancer_gene_census.xls; PMID: 23539594; PMID: 24132290; PMID: 24084849). Pathway enrichment analysis was conducted by metascape software.

### Whole RNA-seq

Whole RNA-seq analysis included lncRNA, mRNA, microRNA and circular RNA (cirRNA). HISAT2 was used to map RNA-seq reads to human reference genome (GRCh38_release95.Homo_sapiens. GRCh38_release95. genome.fa) and assembled with StringTie. lnc RNA was filtered with transcript class code (“i”,“x”,“u”,“o”,“e”), length≥200 bp, Exon≥2 and FPKM ≥ 0.1, and extracted no protein-coding potential intersection from CPC2 (Coding Potential Calculator), CNCI (Coding-Non-Coding Index), CPAT (Coding Potential Assessment Tool) and Pfam database. Neighboring genes (+/-100kb) was defined as lncRNA cis-target gene. Gene expression was transferred to FPKM (fragments per kilobase of transcript per million fragments mapped). Differential expression analysis was conducted with DESeq2, edgeR (fold change ≥1.5 and FDR < 0.05). For cirRNA, BWA was used for human reference genome mapping. CIRI software was used to predict cirRNA and annotated with circBase database, circ2disease and circBank. Specifically, SRPBM standardized strategy and fold change ≥1.5 was adopted for cirRNA expression analysis. For small RNA, sequenced reads (15-35 bp) were included after adapters removal and further rRNA, tRNA, snRNA, snoRNA and repeated sequences were filtered based on Silva, GtRNAdb, Rfam, Repbase database. Unannotated reads were mapped to human reference genome (Homo_sapiens. GRCh38_release95) using Bowtie. Mapped reads were identified with miRBase (v22) database for known miRNA and predicted with miRDeep2 for unannotated miRNA. Specifically, TPM standardized strategy and fold change ≥1.5 were adopted for miRNA expression analysis. Two-sided t-test *p*-value < 0.05.

### Proteomic Data Analysis

Raw data files were analyzed with MaxQuant (v1.5.2.8) for RAW file reading capability. UniProt-GOA database (http://www.ebi.ac.uk/GOA/) was used for Gene Ontology (GO) annotation proteome which converted identified protein ID to UniProt ID and then mapped it to GO IDs. The InterProScan software was used for proteins not annotated based on protein sequence alignment, which was also for proteins domain identification based on InterPro database (http://www.ebi.ac.uk/interpro/). For differential expression analysis, fold change >1.5 (up-regulation) or <1/1.5 (down-regulation) and *p* value < 0.05 was set. Specifically, coefficient of variation (CV) was calculated for T124, T125, P124, and P125 and CV-value < 0.1 was set as the threshold. STRING database (v.10.5) was used for protein-protein interactions analysis accompanied with confidence score >0.7 (high confidence).

### Metabolic Data Analysis

Raw data were converted to the mzXML format by ProteoWizard and processed by R package XCMS (version 3.2) for peak deconvolution, alignment and integration (minfrac 0.5 and cut-off 0.3 respectively). Metabolites identification were conducted with in-house MS2 database. Ropls R package was employed for calculation of OPLS-DA model. Differential metabolites were determined as fold change>1, *p* value < 0.05 and variable importance in the projection >1.

### Statistics and Reproducibility

An independent samples t-test was conducted to evaluate the significance of observed differences between two groups. The *p*-value, typically set at <0.05, was used to determine the likelihood of the observed differences occurring by chance alone. To ensure reproducibility, replicates were utilized under the same experimental conditions. In the analysis, single cells from the same cluster (and/or tumor type) were generally grouped together for comparison purposes.

### Reporting summary

Further information on research design is available in the Nature Portfolio Reporting Summary linked to this article.

### Supplementary information

Supplementary Information

Description of Additional Supplementary Files

Supplementary Data 1

Supplementary Data 2

Reporting summary

## Data Availability

The raw sequence data reported in this paper has been deposited in the Genome Sequence Archive in National Genomics Data Center under the accession number HRA002304, HRA005348, which is accessible at https://ngdc.cncb.ac.cn/gsa-human/browse/. The mass spectrometry proteomics data have been deposited to the ProteomeXchange Consortium via the PRIDE partner repository with the dataset identifier PXD044778. The raw sequence data are available for non-commercial purposes under controlled access because of data privacy laws, and access can be obtained by request to the corresponding authors. Supplementary Data 2 provided the source data for figures.
