# Supplementary material for: Single-cell and spatial architecture of primary liver cancer
Source: Commun Biol. 2023 Nov 20;6:1181. doi: 10.1038/s42003-023-05455-0 (PMC10661180; doi:10.1038/s42003-023-05455-0)
Supplement: Supplementary file 1 — Supplementary Information [file 42003_2023_5455_MOESM1_ESM.pdf]

**Supplementary tables and figures**

**Supplementary Table 1. Demographics and clinicopathologic characteristics of eligible donors for scRNA-seq, spatial transcriptomics and multi-omics.** “-”, negative; “+”, positive; “~”, NA; Tumor capsule: “-”, incomplete or no; “+”, complete; ST, spatial transcriptomics; SC, single-cell RNA-Seq; T, tumor, P, peritumor; B, pre-operative peripheral blood; S, 1 month post-operative peripheral blood; N, metastatic lymph node; “+*”, postoperative (1 month) peripheral blood from P123 was collected twice in 5 day intervals for batch effect detection.

| Characteristic | P121 | P122 | P123 | P124 | P125 | P126 | P127 | P128 | P129 |
|---|---|---|---|---|---|---|---|---|---|
| Age(Y)/Gender | 37/M | 57/M | 63/M | 70/F | 55/M | 66/F | 67/F | 46/M | 68/F |
| Pathology | ICC | ICC | ICC | HCC | HCC | CHC | CHC | HCC | ICC |
| HBsAg/HBeAg/HBeAb/HBcAb | -/-/-/- | -/-/+/+ | +/-/+/+ | -/-/-/+ | +/-/+/+ | -/-/+/+ | -/-/-/- | +/-/+/+ | -/-/-/- |
| Anti-HCV | - | - | - | + | - | - | - | - | - |
| HBV-DNA/HCV-RNA | -/~ | -/~ | -/~ | -/- | +/~ | -/~ | -/~ | +/~ | -/~ |
| AFP(ng/mL) | 3.00 | 1.80 | 4.00 | 678.00 | 2303.00 | 1.20 | 331.00 | 108.00 | 4.30 |
| CEA(ng/mL) | 13.80 | 2.40 | 4.50 | 1.90 | 2.20 | 2.70 | 5.80 | 1.80 | 62.70 |
| CA19-9(U/mL) | 3370.00 | 2.00 | 2.00 | 13.80 | 19.90 | 64.60 | 2.00 | 29.00 | 521.00 |
| AFP-L3(%) | 8.50 | 0.50 | -0.31 | 4.00 | ~ | 0.50 | 97.20 | 3.40 | 27.80 |
| PIVKA-II(mAU/ml) | 27.00 | 44.00 | 11.70 | 4155.00 | 8976.00 | 23.00 | 28.00 | 9305.00 | 23.00 |
| 7-Micro-RNA | + | - | + | - | ~ | ~ | - | + | - |
| TB/DB(umol/L) | 28.2/11.3 | 20.4/6.9 | 4.6/1.3 | 11/3.1 | 12.8/3.9 | 5.9/1.4 | 10.6/3.3 | 15/3.7 | 21.5/4.4 |
| ALT/AST(U/L) | 131/81 | 18/28 | 26/21 | 11/31 | 28/40 | 8/18 | 10/17 | 56/45 | 19/23 |
| PT(s) | 11.6 | 11.0 | 11.1 | 11.4 | 11.2 | 12.6 | 12.1 | 12.6 | 10.7 |
| Tumor number | 1 | 1 | 1 | 1 | 1 | 1 | 1 | 1 | 3 |
| Tumor maximal dimension(cm) | 6 | 11 | 5 | 7 | 8 | 3 | 6 | 10 | 5 |
| Differentiation | II | II | II | II | III | II-III | II-III | II | II |
| VI | - | + | - | - | - | - | - | - | - |
| MVI | - | ++ | - | - | + | - | + | + | - |
| Bile duct embolus | - | - | - | - | - | + | - | - | - |
| Tumor Capsule | + | - | + | + | + | + | - | + | + |
| Lymphatic metastasis | - | - | + | - | - | + | + | - | + |
| Ascite | - | + | - | - | - | - | - | - | + |
| Cirrhosis | - | - | - | + | - | - | - | - | - |
| GS score | G0S0 | G0S0 | G2S2 | G2S4 | G3S3 | G1S1 | G0S0 | G2S3 | G0S0 |
| Child-Pugh score | A | A | A | A | A | A | A | A | A |
| TNM(8th) | IB | II | IV | IB | II | IV | IV | II | IV |
| ST | - | - | - | - | - | - | - | + | + |
| SC(T/P/B/S/N) | +/+/+/-/- | +/+/+/-/- | +/+/+/+*/- | +/+/+/+/- | +/+/+/+/- | +/+/+/-/+ | +/+/+/+/- | -/-/-/-/- | -/-/-/-/- |
| Multi-omics(T/P/N) | +/+ | +/+ | +/+ | +/+ | +/+ | +/+ | +/+ | -/- | -/- |

**Supplementary Table 2. Demographics and clinicopathologic characteristics of external validation tissues.** VI, vascular invasion; MVI, microvascular invasion; TNM, TNM classification based on the AJCC Cancer Staging Manual [8th edition (2017)]; G score, grading of inflammation; S score, staging of fibrosis.

| Characteristic | Variable | HCC(n=89) | ICC(n=48) |
|---|---|---|---|
| Age | years | 55±11 | 60±11 |
| Gender | female/male | 12/77 | 23/25 |
| liver cirrhosis | no/yes | 28/17 | 29/18 |
| Tumor number | 1/>1 | 79/10 | 36/12 |
| Tumor capsule | imcomplete/complete | 26/61 | 40/7 |
| differentiation | poor/moderate/well | 0/71/17 | 8/32/7 |
| VI | no/yes | 87/2 | 45/2 |
| MVI | no/yes | 56/32 | 44/4 |
| Tumor maximal dimension | cm | 5.26±3.27 | 5.72±2.43 |
| Ascite | no/yes | 84/5 | 45/3 |
| TNM | I/II/III/IV | 52/26/10/1 | 28/8/11/1 |
| G score | 0/1/2/3 | 1/24/37/9 | 8/9/12/3 |
| S score | 0/1/2/3/4 | 4/16/12/8/31 | 15/5/5/4/4 |

**Supplementary Figure 1. Unsupervised processing with optimal settings and spatial spot class prediction.** **a,** Effects of clustering based on different K-means, principle components (PCs) and resolution. Normalized mutual information was employed to assess the clustering robustness with different parameters, which set the baseline as components =50, k-means =30, resolution =0.6. **b,** Bar plot showing the number of cells from P121-P127. S, 1 month post-operative peripheral blood; O, postoperative (1 month) peripheral blood from P123 was collected twice in 5 day intervals for batch effect detection. **c,** Heatmap presenting predicted spatial distribution of sub-clusters from the major cell types using MNN and MIA algorithm. The horizontal axis indicates the clusters of ST spots, and the vertical axis indicates the sub-clusters of major cell types. Red color shows higher possibility of cells located in clusters of ST spots. T, tumor zone; P, peritumor zone; J, tumor-peritumor junctional zone; S, stroma zone; F, fatty infiltrated zone; n-F, non-fatty infiltrated zone. **d,** Bar plot showing the proportion of spot class based on different ST slices (left), spatial zone of P129TP1 (middle) and P129TP2 (right). doublet, two or more cells in one spot; singlet, one cell in one spot; uncertain, uncertain in one spot. **e,** Bar plot showing the spot number of doublet with different cell types.

# Supplementary Figure 1.

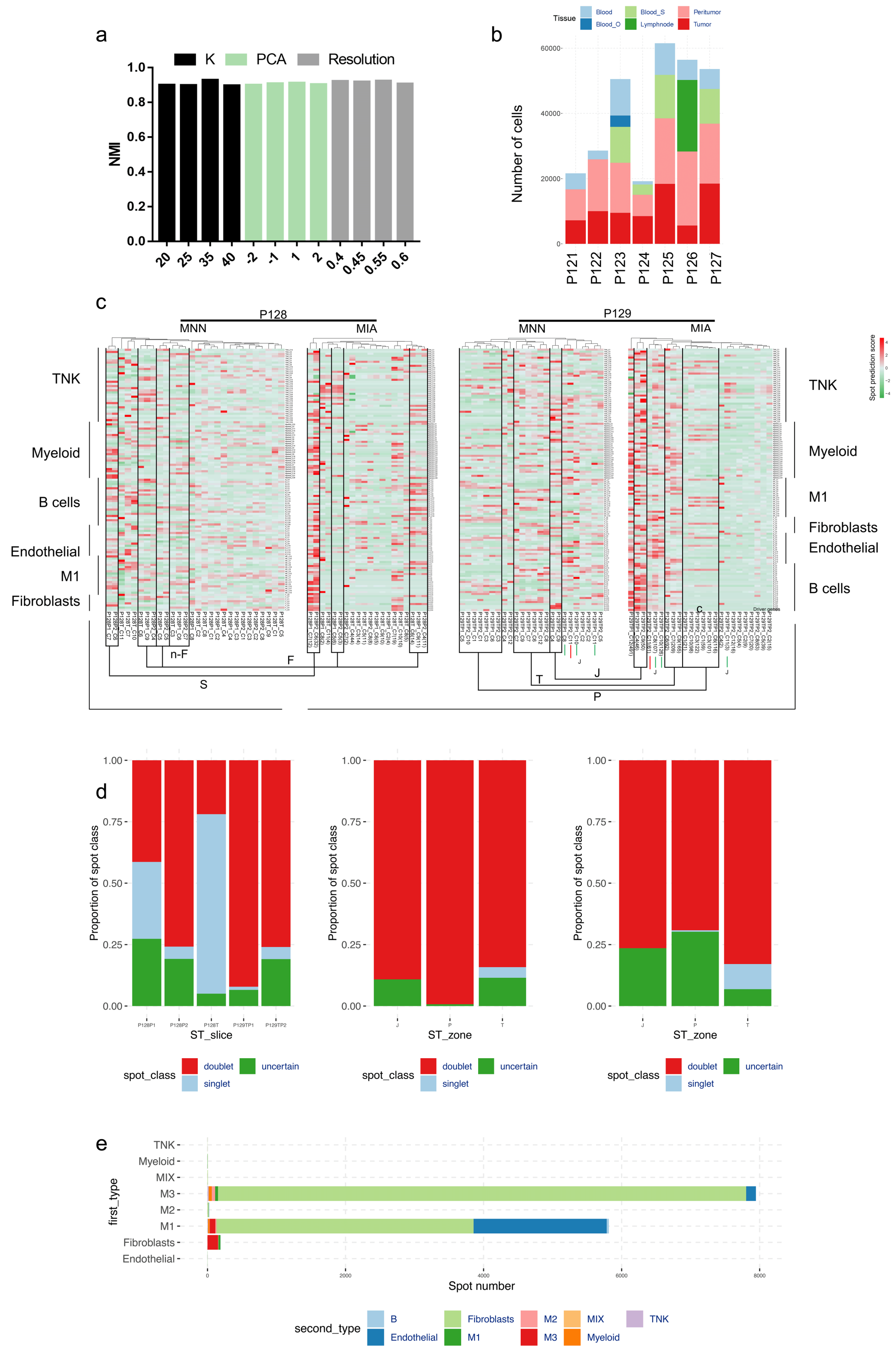

**Supplementary Figure 2. Multi-omics analysis. a,** Heatmap showing the difference of bulk omics (ATAC, RRBS, miRNA, cicrRNA and metabolome) based on samples. T, tumor tissue; P, peritumor tissue. White indicates lower expression and red indicates higher expression. **b,** Heatmap showing the difference of bulk omics (mRNA, lncRNA and proteomics) based on samples. **c,** Bar plot showing the gene variation (left) and variation based on samples (right) using whole-exome sequencing. **d,** Copy number variation and loss of heterozygosity in chromosome (upper), B Allele Frequency in chromosome (middle) and prediction of copy number variation based on SC data (lower). Tumor tissue from Donor 121 (T121) showed more abundant chromosomal alteration compared to peritumor tissue thereof, and displayed the similar chromosomal alteration between bulk and SC data (left). Metastatic lymphnode from Donor 126 (N126) showed the less chromosomal alteration (right). **e,** Heatmap showing the mutation signature based on whole-exome sequencing (left) and the similarity between the mutation signature and cosmic database (right).

**Supplementary Figure 2.**

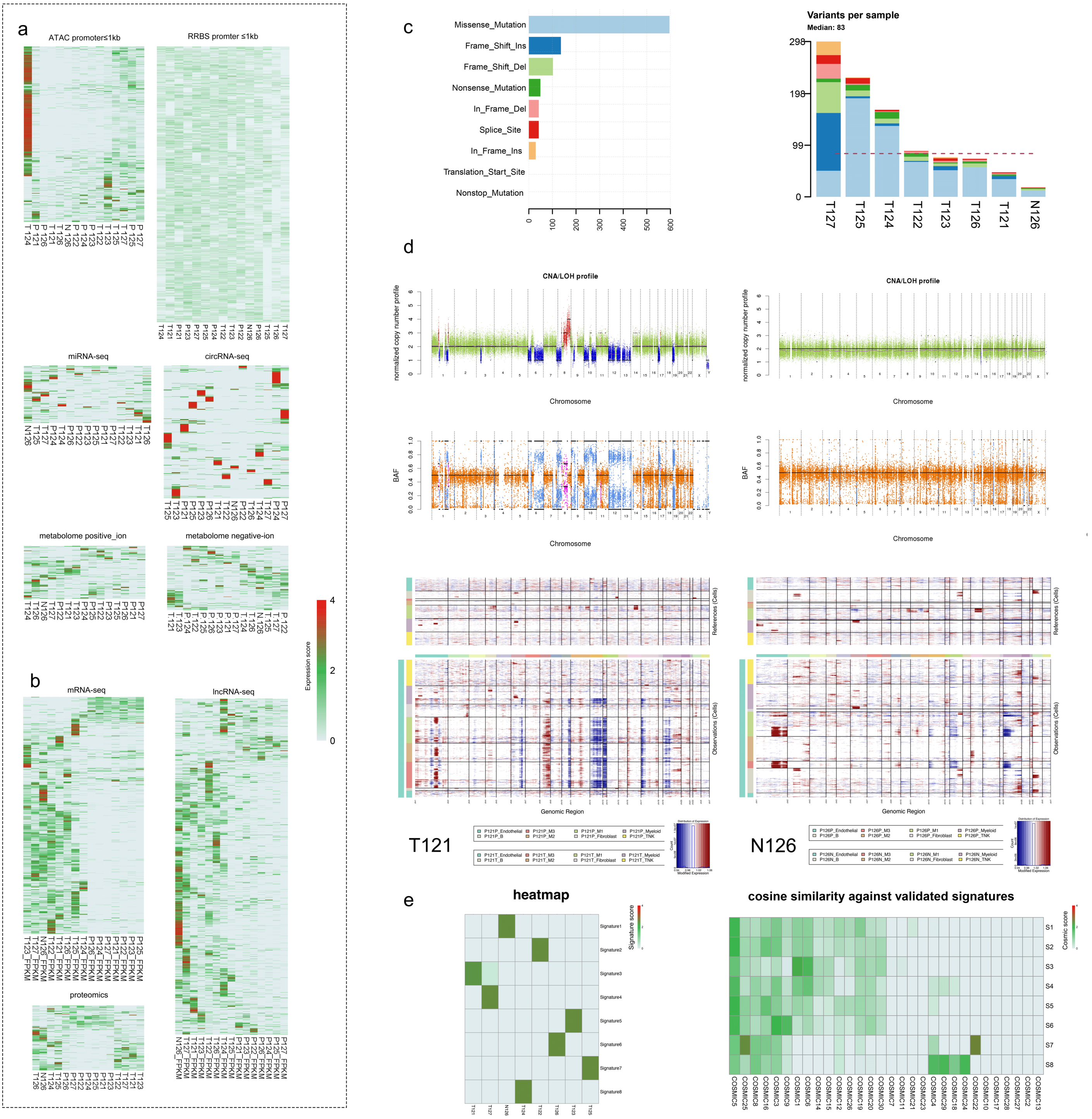

**Supplementary Figure 3**. **Cross analysis based on supra-clusters related to malignant cells (M1, M2 and M3). a,** Principle components analysis of ST spots' clusters based on each ST slice. **b,** The expression of proliferation associated genes based on 3 supra-clusters related to malignant cells (M1, M2 and M3). **c,** UMAP distribution (left) and bar plot (right) of supra-cluster M1 based on tissue. Red color represents cells derived from tumor tissue. **d,** Oncoplot showing the variation of oncogenes detected with whole-exome sequencing. **e,** Stacked violin plot showing the expression of oncogenes in malignant cell sub-clusters of M1. **f,** Metascape pathway enrichment analysis showing the signaling pathway enrichment of differentially expressed genes (bulk tumor vs. bulk peritumor). **g,** CibersortX presenting cell proportion of bulk samples. Cell type of supra-clusters from SC data was used as the reference.

# Supplementary Figure 3.

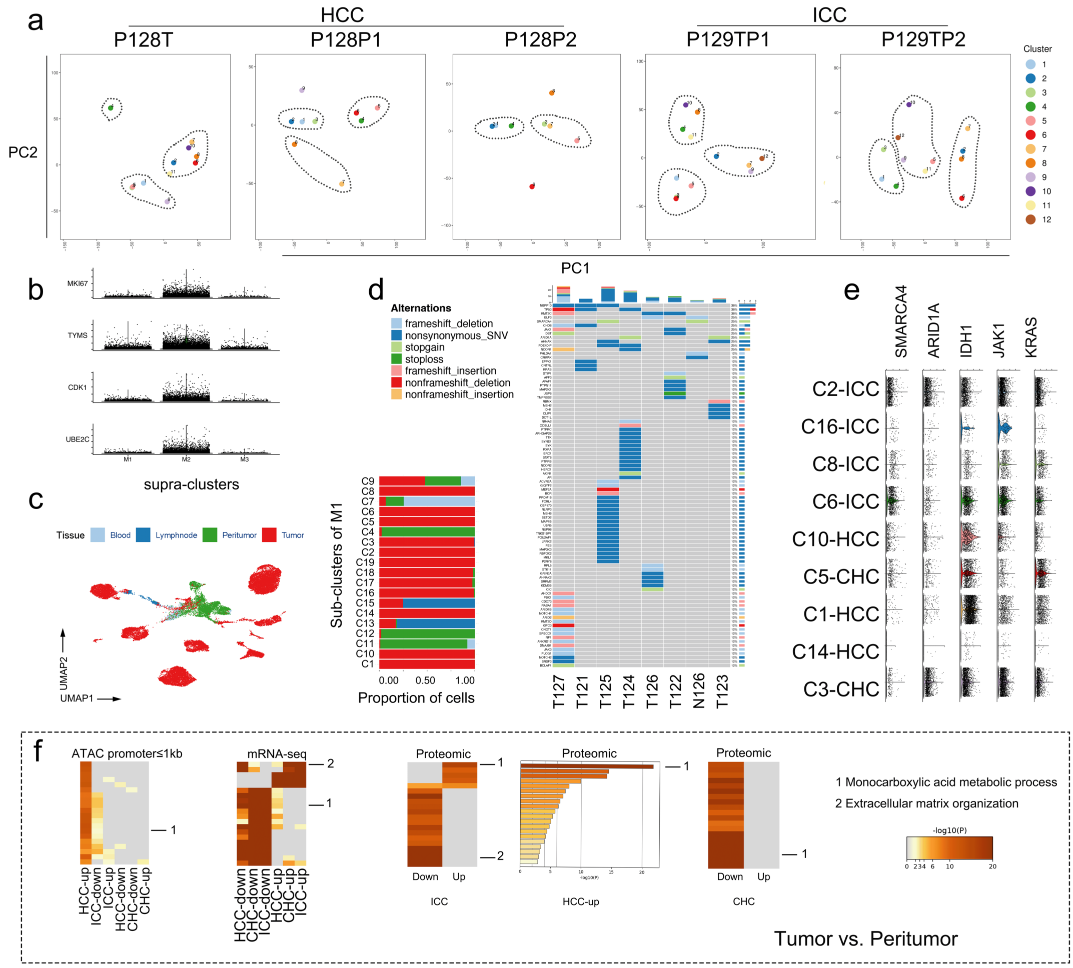


g

bulk samples mRNA

supra-clusters

| Input Sample | TNK | M1 | B | Myeloid | Endothelial | M2 | M3 | Fibroblast | P-value | Correlation | RMSE | Absolute score (sig.score) |
|---|---|---|---|---|---|---|---|---|---|---|---|---|
| P121_FPKM | 0 | 10.233 | 0.089 | 0.755 | 0.739 | 0 | 0.68 | 0.822 | 0 | 0.660 | 0.796 | 13.317 |
| P122_FPKM | 0.028 | 10.01 | 0.019 | 0.729 | 0.761 | 0 | 0.323 | 0.964 | 0 | 0.680 | 0.769 | 12.834 |
| P123_FPKM | 0 | 9.081 | 0.207 | 0.988 | 0.608 | 0 | 0.642 | 1.02 | 0 | 0.671 | 0.772 | 12.546 |
| P124_FPKM | 0 | 10.622 | 0.061 | 0.779 | 0.721 | 0 | 0 | 0.551 | 0 | 0.699 | 0.750 | 12.734 |
| P125_FPKM | 0.162 | 10.606 | 0.162 | 1.357 | 0.921 | 0 | 0.134 | 0.704 | 0 | 0.665 | 0.772 | 14.046 |
| P126_FPKM | 0 | 11.195 | 0.111 | 1.211 | 0.851 | 0 | 0 | 0.389 | 0 | 0.614 | 0.841 | 13.757 |
| P127_FPKM | 0.023 | 10.75 | 0.078 | 1.281 | 0.858 | 0 | 0.285 | 0.653 | 0 | 0.660 | 0.786 | 13.928 |
| T121_FPKM | 0.342 | 0 | 0.061 | 0.711 | 0.097 | 0 | 4.519 | 3.466 | 0 | 0.506 | 0.962 | 9.196 |
| T122_FPKM | 0.141 | 0 | 0.07 | 1.057 | 0.035 | 0 | 6.486 | 1.737 | 0 | 0.708 | 0.767 | 9.526 |
| T123_FPKM | 0.771 | 0 | 0.62 | 0.829 | 0.472 | 0 | 2.991 | 4.103 | 0 | 0.707 | 0.715 | 9.785 |
| T124_FPKM | 0 | 12.106 | 0 | 0 | 0.072 | 0.555 | 0.658 | 0.222 | 0 | 0.897 | 0.464 | 13.614 |
| T125_FPKM | 0 | 9.047 | 0 | 0.633 | 0 | 1.654 | 0 | 0.128 | 0 | 0.881 | 0.475 | 11.461 |
| T126_FPKM | 0 | 3.017 | 0.195 | 1.91 | 0.069 | 0 | 2.916 | 1.546 | 0 | 0.390 | 1.020 | 9.652 |
| T127_FPKM | 0 | 0 | 0.677 | 1.214 | 0.041 | 0.957 | 2.931 | 1.212 | 0 | 0.592 | 0.818 | 7.033 |
| N126_FPKM | 0.589 | 0.621 | 2.315 | 1.488 | 0.158 | 0.352 | 1.126 | 0.654 | 0 | 0.484 | 0.876 | 7.302 |

CibersortX_with sc-RNA reference

● low polar ● high polar

**Supplementary Figure 4. Spatial distribution prediction and cell-cell interaction analysis. a,** Spatial distribution prediction of CAFs in P129 ST slices using MIA and MNN algorithm. Red color shows higher possibility of cells located in the area. **b,** Radar chart showing the cell-cell interaction between fibroblast supra-cluster and other supra-clusters. **c,** Circos plot showing the ligand-receptor pairs of cell-cell interaction between fibroblast sub-cluster and other cell sub-clusters.

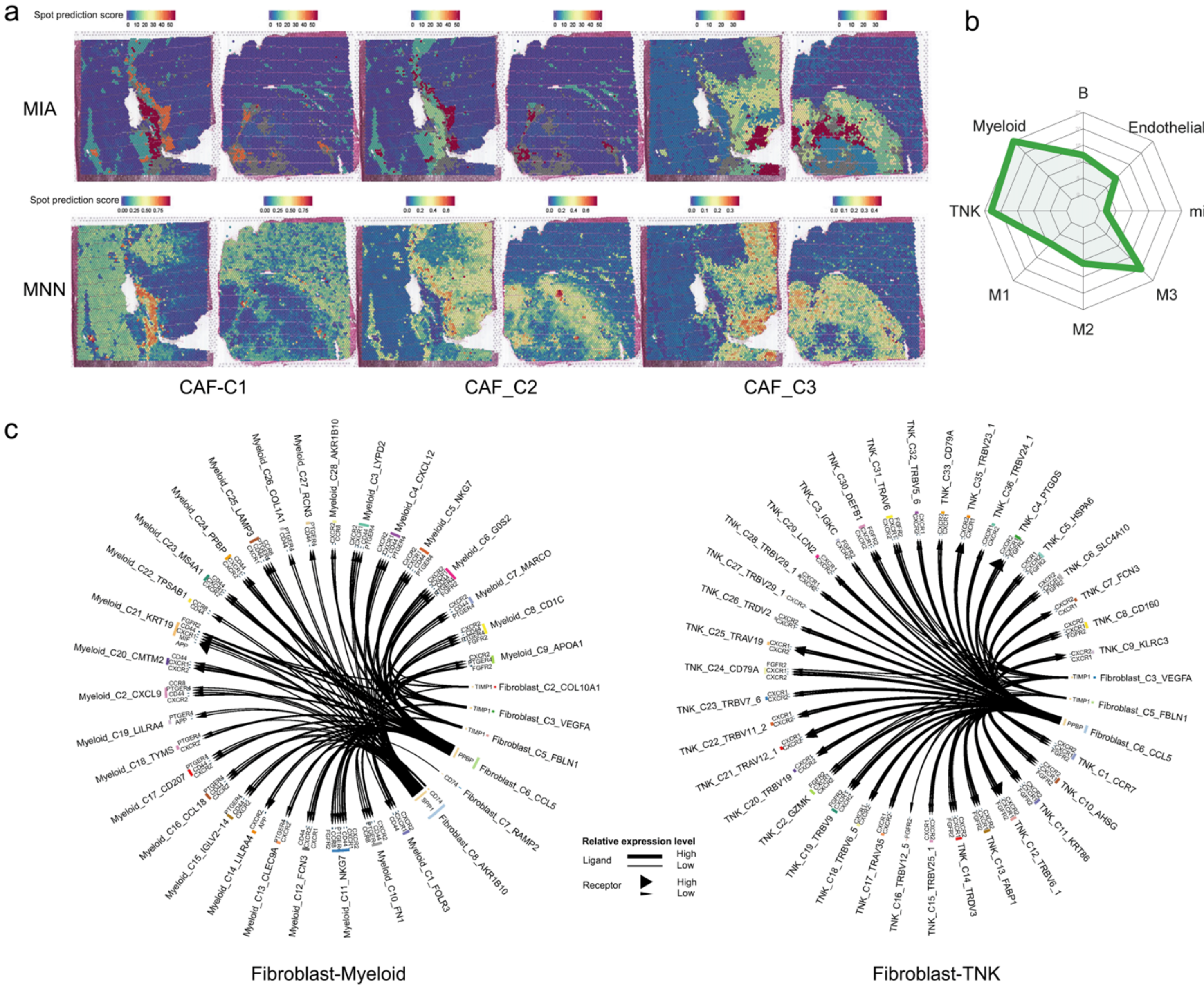

**Supplementary Figure 5**. **Cross-validation of endothelial cells. a,** Collection workflow of $CD45^{-}CD31^{+}$ endothelial cells using magnetic cell separation. **b,** inferCNV analysis showing the copy number variation of endothelial cells from SC data based on tissue, and presenting less chromosomal alteration. **c,** Whole-exome sequencing was performed to detect copy number variations. Horizontal axis indicates the location of chromosome, and vertical axis indicates the abundance of copy number variations. B_vs_P, peripheral blood cells vs. peritumor-derived $CD45^{-}CD31^{+}$ endothelial cells (upper); T_vs_P, tumor-derived $CD45^{-}CD31^{+}$ endothelial cells vs. peritumor-derived $CD45^{-}CD31^{+}$ endothelial cells (middle); J_vs_P, $CD45^{-}CD31^{+}$ endothelial cells derived from tumor-peritumor junctional zone vs. peritumor-derived $CD45^{-}CD31^{+}$endothelial cells (lower). **d,** Radar chart showing the cell-cell interaction between endothelial cell supra-cluster and other supra-clusters. **e,** Circos plot showing the ligand-receptor pairs of cell-cell interaction between endothelial cell sub-cluster and fibroblast sub-clusters. **f,** Experiment controls of RNA-scope ISH staining. The staining marker was recommended by the protocol (POLR2A, PPIB and UBC). Positive controls (upper), negative controls (lower). Scale bars, 5μm.

**Supplementary Figure 5.**

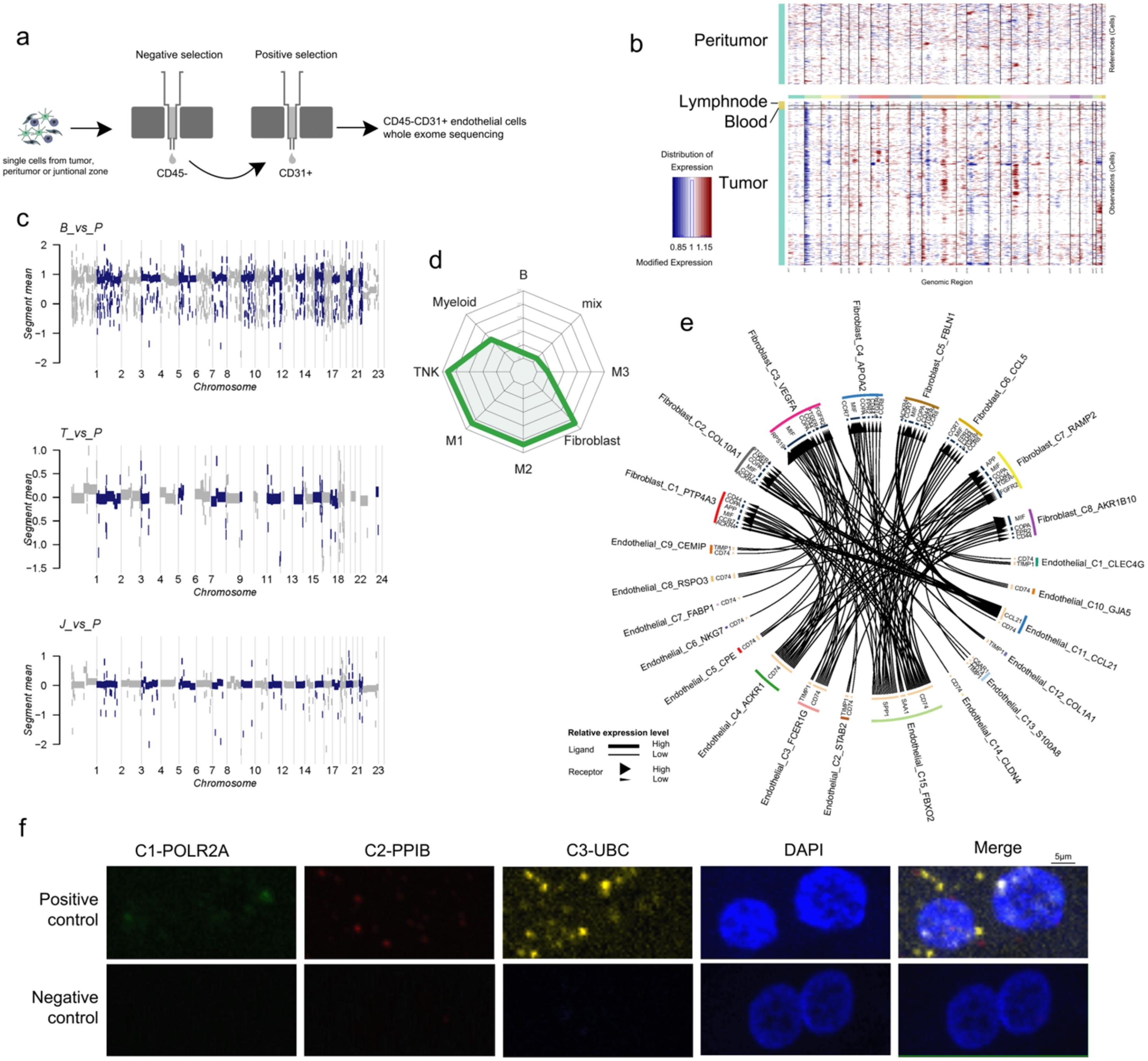

**Supplementary Note 1. ICC showed richer clonal diversity of TCR repertoire among 3 primary liver cancer types.**

To enable a systematic comparison of immune cells among different types of PLC, we re-clustered 9 TNK, 2 B and 8 myeloid cell supra-clusters respectively, obtaining 31 sub-clusters of TNK cells, 22 sub-clusters of B cells, 23 sub-clusters of myeloid cells and 13 mixed cell sub-clusters, which made comparison more detailed, and made it more accessible to distinguish mixed cells (Supplementary Figure 6a). In proportion analysis, there is lower proportion of T cells infiltrating into the tumor tissue (Supplementary Figure 6b), consistent with prior studies[1,2].

Of 31 TNK sub-clusters, we identified 7 CD4$^+$ T cell sub-clusters, 15 CD8$^+$ T cell sub-clusters, 2 CD3$^+$CD4$^-$CD8$^-$ T cell sub-clusters, 1 mucosal-associated invariant T cell (MAIT) sub-cluster, 3 NK cell sub-clusters and 3 NKT cell sub-clusters (Supplementary Figure 6c), of which cell diversity was consistent with prior study[2]. We found 3 of 7 CD4$^+$ T cell sub-clusters (C5, C10 and C13) and only 1 of 15 CD8$^+$ T sub-clusters (C16) were tumor-derived clusters. Those tumor-derived T cells showed a higher proliferation ratio and greater patient heterogeneity. We observed that 5 CD8$^+$ and 4 CD4$^+$ T cell sub-clusters were mainly derived from HCC, and 3 CD8$^+$ T cell sub-clusters from CHC, however, there was no specific ICC-derived sub-clusters (Supplementary Figure 7a). To gain deeper insight into this result, we analyzed the clonal diversity of T cell receptor (TCR) repertoire which was simultaneously sequenced corresponding to our 5' scRNA-seq libraries, hinting the clonotypes of T cells in ICC more diverse than those in HCC ($p<0.05$) and CHC ($p>0.0.5$). This might explain why ICC lacks its derived heterogeneous sub-clusters (Supplementary Figure 6d).

We next employed pseudotime trajectory to construct the possible developmental trajectory of CD4$^+$ and CD8$^+$ T cells. Generally, it displayed a process from naïve to effector to exhausted T cells in pseudotime trajectory, in agreement with prior studies[2] (Supplementary Figure 6e, Supplementary Figure 7b). Of note, we did not find sub-clusters of naïve CD8$^+$ T cells, partly due to our strategy of unsupervised cell collection. However, we identified 7 CD8$^+$ T cell sub-clusters (C2, C17, C18, C23, C32, C36 and C37) in the middle of trajectory, consistent with their intermediate states of functionality (Supplementary Figure 6e). As a representative of them, CD8$^+$ C2 marked with GZMK was the largest intermediate-state sub-cluster (11.35% of all TNK supra-clusters, 15663/137949) that was universal in 3 types of PLCs across all patients. It expressed both exhausted markers (PDCD1 and LAG3) and cytotoxic markers (GZMA and GZMK), in line with previous reports on HCC[2] (Supplementary Figure 6c). Clonal analysis, which focused on identical TCRs from the same ancestry but shared by different sub-clusters, revealed that CD8$^+$GZMK$^+$ C2, aligned with its intermediate state, presented a relatively dynamic state of shared TCR sequences with other sub-clusters. For instance, C2 shared TCRs with effector memory T cell sub-clusters C19 (in 1,111 cells from both C2 and C19) and C20 (1,235 cells), and with intermediate sub-clusters C18 (1,316 cells) and C23 (437 cells) (Supplementary Figure 6f). To further

investigate whether intermediate-state functionality of C2 might connect to its spatial distribution pattern, we employed MNN and MIA algorithm, and it suggested that intermediate-state C2 were more likely to be located in J and S zones of P129TP1 and P129TP2 (Supplementary Figure 7c). These results indicate that the pervading intermediate-state cell populations in PLCs might link with their spatial distribution, and play a significant role in cell transition. Further exploration is needed in terms of their molecular mechanism and therapeutic potential.

We also identified 3 NK sub-clusters (C4, C8 and C11) and 3 NKT sub-clusters (C7, C9 and C14). NK cells performed natural killer cell mediated cytotoxicity while NKT cells performed acute inflammatory response, consistent with prior study[3] (Supplementary Figure 6c, Supplementary Figure 7d). Of note, they were mainly derived from peritumor tissues except C11 from tumor tissues (Supplementary Figure 7a). In addition, MAIT sub-cluster (C6 marked with SLC4A10)[2], mainly distributed in the peritumor, was found existing in all 3 types of PLCs (Supplementary Figure 7a).

In terms of B cells (Supplementary Figure 6a.g), 22 related sub-clusters were assigned to 9 sub-clusters of memory B cells, featuring in activation of adaptive immune system, and 13 sub-clusters of plasma B cells, featuring in complement activation and protein processing in endoplasmic reticulum, as previously reported[2]. Through clonal analysis with BCR sequences, the majority of memory B cells was found to be monoclonal, while the majority of plasma B cells to be polyclonal. Moreover, abundant identical BCRs between memory and plasma B cells hinted dynamic cell type switching among them (Supplementary Figure 6h.i, Supplementary Figure 7e). Notably, we observed the largest sub-cluster of B cells (C1, 4,450 cells) identified as $STAG3^{+}CD27^{-}$ memory B cells, and pseudotime trajectory displayed cells from C1 were located in the middle of trajectory, with memory and plasma B cells at opposite ends (Supplementary Figure 7g). Meanwhile, C1 presented multi-directional flows toward other sub-clusters in RNA velocity (Supplementary Figure 6i). These results indicate that these B cells are at the early stage of cell differentiation. C1 in a monoclonal state displayed higher clonal diversity than plasma B cell and other memory B cell sub-clusters, partly consistent with the recently reported $CD45RB^{+}CD27^{-}$ early memory B cell population that had intermediate levels of BCRs diversity and mutational burden[4]. We then investigated whether C1 might relate to its spatial distribution. C1 was found mainly derived from the metastatic lymph node (P126N, 98%), and a small proportion of C1 was collected from tumor and peritumor tissues, but rare from peripheral blood. The metascape enrichment analysis found that C1 was related to lymphocyte activation and active metabolism such as peptide chain elongation (Supplementary Figure 7h). These indicate that certain cells of C1 might be affected by local stimuli residing in TME. Together, these results suggest that C1 as a sub-cluster with huge cell populations more closely links to early memory B cells, retaining the potential for cell transition to effector cells and serving as a potential target for treatment.

With respect to myeloid cells, 23 related sub-clusters were assigned into sub-clusters of

monocytes, macrophages and dendritic cells (DCs) (Supplementary Figure 6a). Four of 9 macrophage sub-clusters exhibited distinct heterogeneity at both tumoral and patient levels, identified as tumor associated macrophages (TAMs; C2, C9, C10 and C16). Consistent with previous reports[5], monocytes and normal macrophages (C4, C7, C12 and C27) presented a developmental continuum in RNA velocity, while these TAMs displayed distinct features of global transcriptomic maturation process (Supplementary Figure 7i). Of note, there was a macrophage sub-cluster (C6) derived from tumor and peritumor tissues in between monocytes and TAMs in UMAP, expressing chemoattractant cytokine genes CXCL2 and CXCL8, and related to inflammatory inhibition reaction like IL10, IL17 cytokine signaling pathway, suggesting C6 is a population of intermediate-state macrophages (Supplementary Figure 6a, Supplementary Figure 7j). DCs presented a relatively separate distribution in UMAP (Supplementary Figure 6a), which included classical DC2 (cDC2; C8 and C17), conventional DC1 (cDC1; C13), plasmacytoid DCs (pDCs; C14 and C19) and other DCs (C15, C23 and C25). C25 was a sub-cluster marked with LAMP3, which was recently reported in HCC with features of maturation and migration capacity toward lymph node[5], and we further observed it abundant in tissues of tumor, peritumor and metastatic lymph-nodes across all 3 types of PLCs (Supplementary Figure 7k). Besides, we noticed a highly proliferative sub-cluster (C18) mainly derived from tumor tissues (Supplementary Figure 6j). It was also predicted to be located in T zone through MNN and MIA (Supplementary Figure 6k). Cells from C18 highly expressed TYMS, and this gene is critical for DNA replication and repair, and of interest as a target for cancer chemotherapeutic agents[6]. This suggests TYMS-targeted therapy might function in tumors through these highly proliferative cells.

To investigate immune alteration after tumor burden removal, we clustered 69,750 cells from both peripheral blood samples at 1-month post-operation and matched pre-operative blood samples of 4 patients (P123, P124, P125 and P127), obtaining 25 clusters of TNK cells, 3 clusters of B cells, and 4 clusters of myeloid cells (Supplementary Figure 6a). We observed naïve T cells and memory B cells decreased, while plasma B cells (C17) and an NKT-like cell cluster (C10) increased in post-operative blood samples compared to those in pre-operative ones, suggesting immune cell composition in peripheral blood was reshuffled by tumor burden removal, with increased effector cells and decreased naïve cells (Supplementary Figure 6l). Taken together, our data suggest immune cells do not show significant discrepancy over 3 PLC types, however, T cells appear to have richer clone diversity in ICC and to be more patient-specific heterogeneous in HCC. We observed abundant $CD8^+$ intermediate-state T cells, and their states might connect to their spatial distribution in J zones. We also observed 2 highly proliferative sub-clusters that contain potential value for targeted therapies.

**Supplementary Figure 6. Immune cells in PLCs. a,** UMAP distribution of re-clustered TNK cells (T, NK and NKT cells), B cells, myeloid cells, and cells from both

peripheral blood samples at 1-month post-operation and matched pre-operative blood samples. **b,** Box plot presenting the proportion of T cells, B cells and myeloid cells in PLC types separated with tumor types and tissue types. Two-sided t-test: ns, no significant; *, $p < 0.05$. **c,** Expression heatmap of selected function-associated genes in each TNK sub-cluster. MAIT, mucosal-associated invariant T cell. **d,** Box plot showing clonal diversity of T cell receptor repertoire based on TRB. TRB, T cell receptor beta chain. Two-sided t-test: ns, no significant; *, $p < 0.05$. **e,** Pseudotime trajectory of $CD8^+$ T cells. Red dots present $CD8^+GMK^+$ intermediate-state sub-cluster C2. **f,** Counts of cells with identical TCRs between $CD8^+$ T cell sub-clusters. The line weights present shared cell numbers. TCRs, T cell receptor chains; EM, effector memory T cells; EMRA, effector memory recently activated T cells; intermediate, intermediate-state T cells. **g,** Bar plot presenting the proportion of cell cycle phase based on each B cell sub-cluster. Memory, memory B cells; plasma, plasma B cells. **h,** Counts of cells with identical BCRs between B cell sub-clusters. BCRs, B cell receptor chains. The line weights present shared cell numbers. **i,** RNA velocity visualization of dynamic flows among B cell sub-clusters. Arrows show the directions. **j,** Bar plot presenting the proportion of cell cycle phase based on each myeloid cell sub-cluster. **k,** Spatial distribution prediction of $TYMS^+$ C18 myeloid cells in P129 ST slices using MIA and MNN algorithm. Red color shows higher possibility of cells located in the area. **l,** Bar plot presenting the proportion of cells between pre-operative and post-operative peripheral blood based on each sub-cluster. The dash line highlights the proportion ratio of 0.50.

# Supplementary Figure 6.

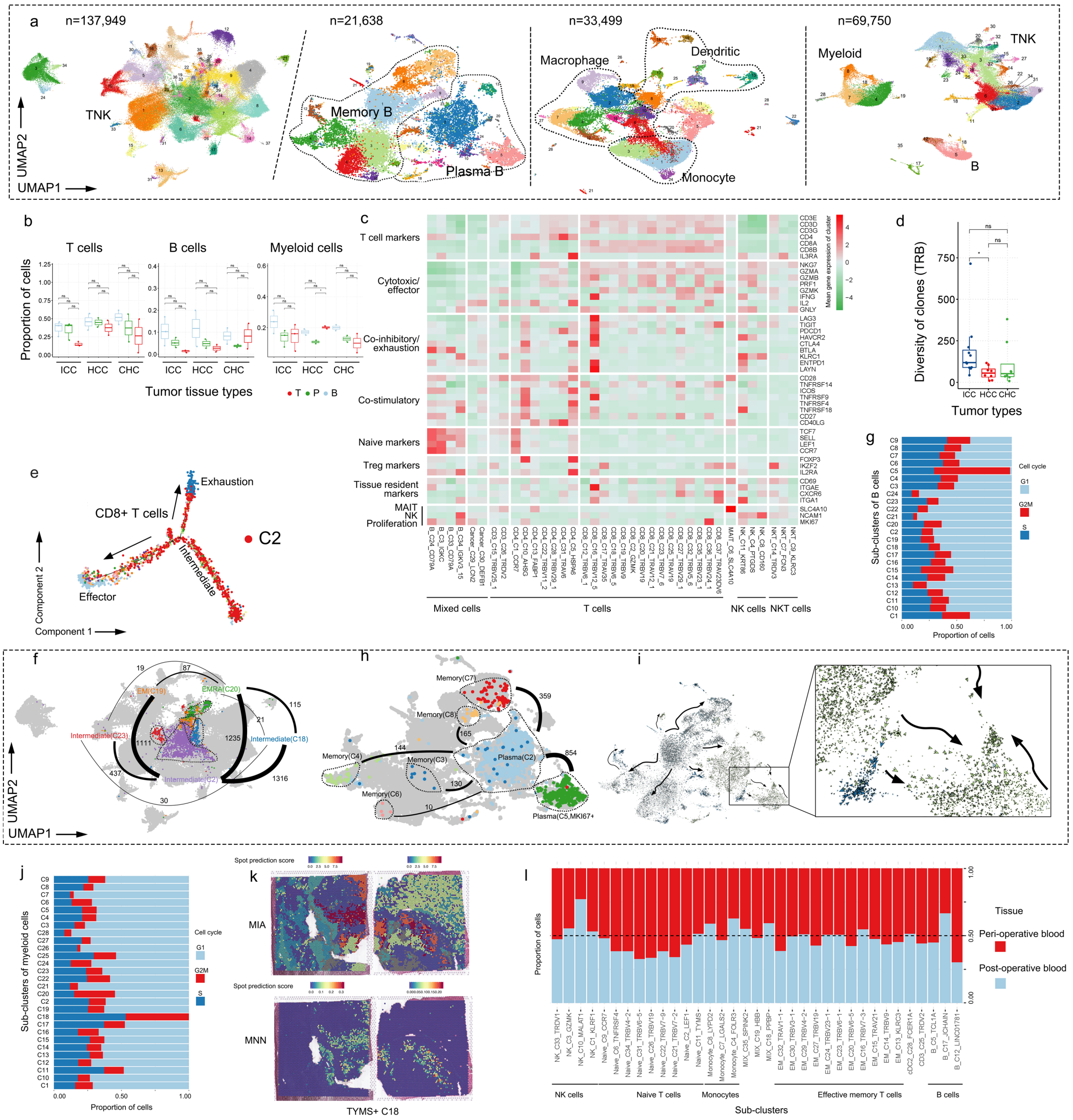

**Supplementary Figure 7. Cross analysis of immune cells. a,** Bar plot showing the proportion of TNK sub-clusters based on tissue and patient. **b,** Pseudotime trajectory of $CD4^+$ T cells. **c,** Spatial distribution prediction of intermediate-state $GZMK^+CD8^+$ T cells (C2) in P129 ST slices using MIA and MNN algorithm. Red color shows higher possibility of cells located in the area. **d,** Metascape pathway enrichment analysis based on differentially expressed genes between NK and NKT cells. **e,** Bar plot showing the proportion of cells based on B cell sub-clusters. N1, one-cell clonotype (monoclonal); N2, 2-cell clonotype (biclonal); N3, clonotype with more than 2 cells (polyclonal). **f,** GSVA of B cell sub-clusters. **g,** Pseudotime trajectory of B cells. **h,** Metascape pathway enrichment analysis based on differentially expressed genes of B cell sub-cluster (C1) compared with other B cells. **i,** RNA velocity visualization of dynamic flows among myeloid cells. Arrows show the directions. **j,** Metascape pathway enrichment analysis based on differentially expressed genes of myeloid cell sub-cluster (C6). **k,** Bar plot of myeloid cell sub-clusters based on tissue. Lower, inside and upper horizontal line of the box plot indicate first quartile, median, and third quartile, separately.

**Supplementary Figure 7.**

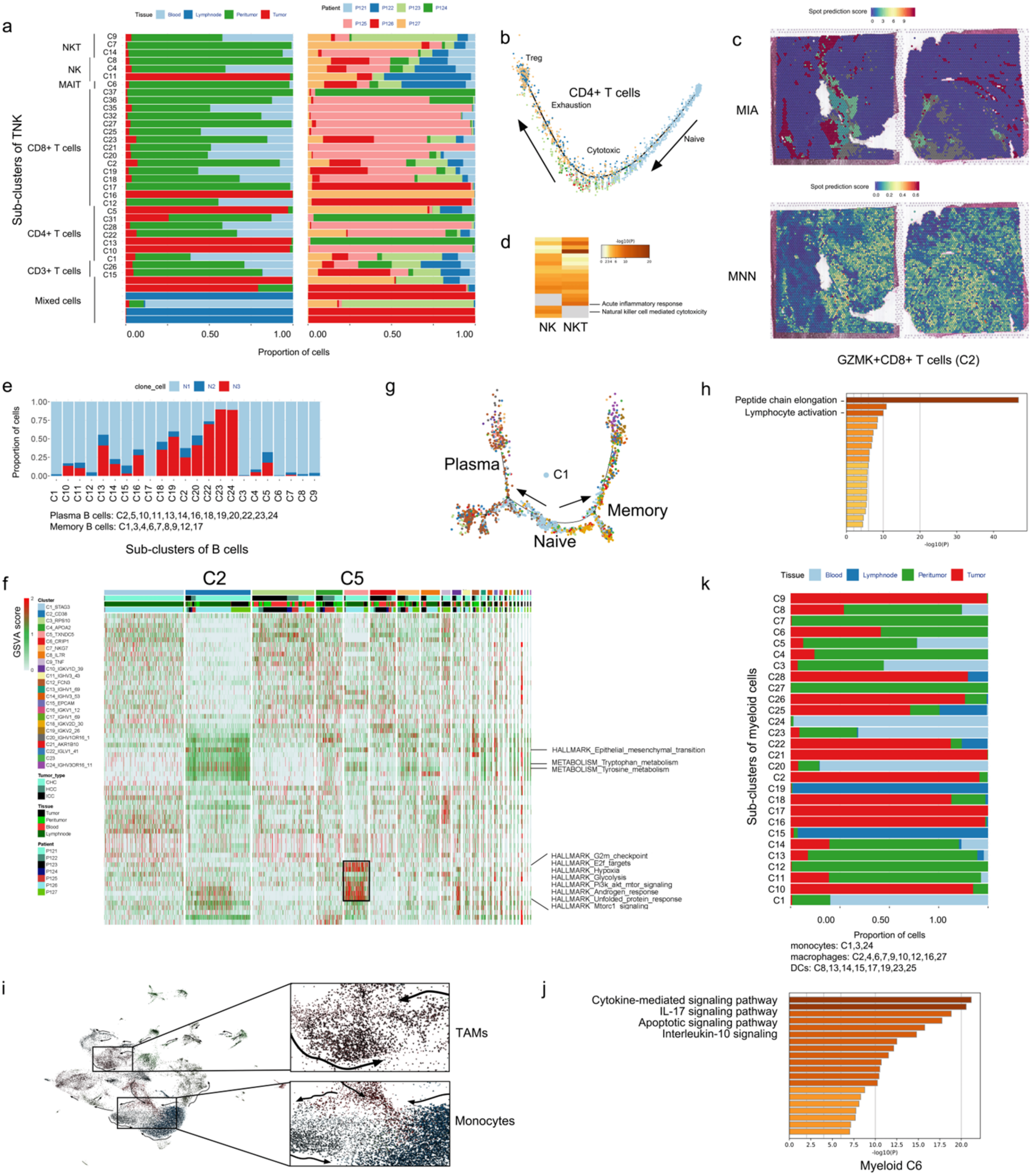

## Supplementary References.


1 Zhang, M. *et al.* Single-cell transcriptomic architecture and intercellular crosstalk of human intrahepatic cholangiocarcinoma. *J Hepatol* **73**, 1118-1130, doi:10.1016/j.jhep.2020.05.039 (2020).

2 Zheng, C. *et al.* Landscape of Infiltrating T Cells in Liver Cancer Revealed by Single-Cell Sequencing. *Cell* **169**, 1342-1356 e1316, doi:10.1016/j.cell.2017.05.035 (2017).

3 MacParland, S. A. *et al.* Single cell RNA sequencing of human liver reveals distinct intrahepatic macrophage populations. *Nat Commun* **9**, 4383, doi:10.1038/s41467-018-06318-7 (2018).

4 Glass, D. R. *et al.* An Integrated Multi-omic Single-Cell Atlas of Human B Cell Identity. *Immunity* **53**, 217-232 e215, doi:10.1016/j.immuni.2020.06.013 (2020).

5 Zhang, Q. *et al.* Landscape and Dynamics of Single Immune Cells in Hepatocellular Carcinoma. *Cell* **179**, 829-845 e820, doi:10.1016/j.cell.2019.10.003 (2019).

6 Demetriadou, C. *et al.* Histone N-terminal acetyltransferase NAA40 links one-carbon metabolism to chemoresistance. *Oncogene*, doi:10.1038/s41388-021-02113-9 (2021).
