# Supplementary material for: Single-cell and spatial architecture of primary liver cancer
Source: Commun Biol. 2023 Nov 20;6:1181. doi: 10.1038/s42003-023-05455-0 (PMC10661180; doi:10.1038/s42003-023-05455-0)
Supplement: Supplementary file 2 — Description of Additional Supplementary Files [file 42003_2023_5455_MOESM2_ESM.pdf]

Description of Additional Supplementary Files

File name: Supplementary Data 1

Description: Profiling of single-cell and spatial transcriptomic cohort. Doublet ratio of single-cell samples.

File name: Supplementary Data 2

Description: Source data underlying the graphs and charts presented in the main figures.
