# Supplementary material for: Single-cell and spatial architecture of primary liver cancer
Source: Commun Biol. 2023 Nov 20;6:1181. doi: 10.1038/s42003-023-05455-0 (PMC10661180; doi:10.1038/s42003-023-05455-0)
Supplement: Supplementary file 5 — Reporting summary [file 42003_2023_5455_MOESM5_ESM.pdf]

nature portfolio

Corresponding author(s): Shuangjian Qiu

Last updated by author(s): Sep 20, 2023

Reporting Summary

Nature Portfolio wishes to improve the reproducibility of the work that we publish. This form provides structure for consistency and transparency in reporting. For further information on Nature Portfolio policies, see our Editorial Policies and the Editorial Policy Checklist.

Statistics

For all statistical analyses, confirm that the following items are present in the figure legend, table legend, main text, or Methods section.

n/a	Confirmed	
☐	☒	The exact sample size (*n*) for each experimental group/condition, given as a discrete number and unit of measurement
☐	☒	A statement on whether measurements were taken from distinct samples or whether the same sample was measured repeatedly
☐	☒	The statistical test(s) used AND whether they are one- or two-sided<br>*Only common tests should be described solely by name; describe more complex techniques in the Methods section.*
☒	☐	A description of all covariates tested
☒	☐	A description of any assumptions or corrections, such as tests of normality and adjustment for multiple comparisons
☐	☒	A full description of the statistical parameters including central tendency (e.g. means) or other basic estimates (e.g. regression coefficient) AND variation (e.g. standard deviation) or associated estimates of uncertainty (e.g. confidence intervals)
☐	☒	For null hypothesis testing, the test statistic (e.g. *F*, *t*, *r*) with confidence intervals, effect sizes, degrees of freedom and *P* value noted<br>*Give P values as exact values whenever suitable.*
☒	☐	For Bayesian analysis, information on the choice of priors and Markov chain Monte Carlo settings
☒	☐	For hierarchical and complex designs, identification of the appropriate level for tests and full reporting of outcomes
☐	☒	Estimates of effect sizes (e.g. Cohen's *d*, Pearson's *r*), indicating how they were calculated

Our web collection on statistics for biologists contains articles on many of the points above.

Software and code

Policy information about availability of computer code

Data collection	Single-cell and V(D)J library were generated using 10X Chromium 5′ gene expression single cell reagent v1.0 kit (10X Genomics). The Spatial slide was remounted to The Visium Spatial Gene Expression Slide (10x Genomics). Sequencing was performed on a Nova-seq 6000 (Illumina). The bulk samples process, including assay for transposase-accessible chromatin with high throughput sequencing (ATAC-seq) and reduced representation bisulfite sequencing (RRBS) in epigenomics, whole-exome sequencing (WES) in genomics, whole RNA-seq in transcriptomics, isobaric tandem mass tags (TMT)-based global proteomics, and LC-MS in metabolomics, was provided in "Methods" section in details.
Data analysis	Detailed information of data analysis were provided in "Methods" section

For manuscripts utilizing custom algorithms or software that are central to the research but not yet described in published literature, software must be made available to editors and reviewers. We strongly encourage code deposition in a community repository (e.g. GitHub). See the Nature Portfolio guidelines for submitting code & software for further information.

Data

Policy information about availability of data

All manuscripts must include a data availability statement. This statement should provide the following information, where applicable:

- Accession codes, unique identifiers, or web links for publicly available datasets
- A description of any restrictions on data availability
- For clinical datasets or third party data, please ensure that the statement adheres to our policy

The raw sequence data reported in this paper has been deposited in the Genome Sequence Archive in National Genomics Data Center under the accession number HRA002304, HRA005348, which is accessible at https://ngdc.cncb.ac.cn/gsa-human/browse/. The mass spectrometry proteomics data have been deposited to the ProteomeXchange Consortium via the PRIDE partner repository with the dataset identifier PXD044778. The raw sequence data are available for non-commercial purposes under controlled access because of data privacy laws, and access can be obtained by request to the corresponding authors.

Human research participants

Policy information about studies involving human research participants and Sex and Gender in Research.

Reporting on sex and gender	Demographics and clinicopathologic characteristics of eligible donors were reported in Supplementary tables and figures: Supplementary Table1
Population characteristics	Demographics and clinicopathologic characteristics of eligible donors were reported in Supplementary tables and figures: Supplementary Table1
Recruitment	The research was approved by Ethics Committee of Zhongshan Hospital, Fudan University (approval number B2019-216R). Informed consent was obtained from each patient for collection and research of surgically removed liver and peripheral blood samples. All employed protocols in this study abided by the 'Regulations on the Management of Human Genetic Resources' administered by The Ministry of Science and Technology (approval number 2021BAT0574, 2022BAT1853).
Ethics oversight	The research was approved by Ethics Committee of Zhongshan Hospital, Fudan University (approval number B2019-216R).

Note that full information on the approval of the study protocol must also be provided in the manuscript.

Field-specific reporting

Please select the one below that is the best fit for your research. If you are not sure, read the appropriate sections before making your selection.

☒ Life sciences ☐ Behavioural & social sciences ☐ Ecological, evolutionary & environmental sciences

For a reference copy of the document with all sections, see nature.com/documents/nr-reporting-summary-flat.pdf

Life sciences study design

All studies must disclose on these points even when the disclosure is negative.

Sample size	No sample size calculation was performed to pre-determine sample sizes. Sample size for scRNA-seq was determined by the availability of PLC patient samples. SC dataset comprises 289,156 high-fidelity sequenced cells (247,515 from 22 peri-operative samples) and 110,013 V(D)J-detected cells (SC immune repertoire, 91,152 TCRs and 18,861 BCRs). A total of 21,571 ST spots were obtained from P128T, P128P1, P128P2, P129TP1 and P129TP2, at a median depth of 10,995 UMIs/spot and 3,092 genes/spot.
Data exclusions	Low quality data after quality control were excluded from the SC and ST datasets. The data were analyzed with unsupervised processing.
Replication	All these donors in SC cohort contain matched tumor, peritumor, peripheral blood respectively and 4 patients contain one-month postoperative peripheral blood and one patient contains metastatic lymph node. Eventually, we collected 27 samples for scRNA-seq, and 5 slices for ST derived from one HCC and one ICC donor. The 3 types of PLC relate to 2-3 donors respectively, which reduces the bias to a certain degree when PLC entities are horizontally compared. Regarding these 5 slices for ST, internal repetitions were conducted to reduce possible bias, for instance the 2 peritumor serial specimens (P128P1 and P128P2), and primary lesion and sub-lesion (P129TP1 and P129TP2).
Randomization	For computational efficiency, the samples were randomly down-sized to 5000 cells for those groups over 10000.
Blinding	For SC and ST data analysis, ,there was no need for blinding procedure due to no treatment was done. RNAscope ISH and fluorescent mIHC staining score was assessed by two independent pathologists who were blinded to group allocation and patients' clinicopathological data during data analysis.

Reporting for specific materials, systems and methods

We require information from authors about some types of materials, experimental systems and methods used in many studies. Here, indicate whether each material, system or method listed is relevant to your study. If you are not sure if a list item applies to your research, read the appropriate section before selecting a response.

Materials & experimental systems

n/a	Involved in the study
☐	☒ Antibodies
☒	☐ Eukaryotic cell lines
☒	☐ Palaeontology and archaeology
☒	☐ Animals and other organisms
☒	☐ Clinical data
☒	☐ Dual use research of concern

Methods

n/a	Involved in the study
☒	☐ ChIP-seq
☒	☐ Flow cytometry
☒	☐ MRI-based neuroimaging

Antibodies

Antibodies used

Information of the antibodies used in the study.
1. SMA (1:200), Cat# BM0002, boster;
2. Vimentin(1:1000), Cat# 10366-1-AP, PTG;
3. Fibronectin(1:500), Cat# 66042-1-Ig, PTG;

Validation

All antibodies used are commercially available and their manufacturers provided their validation documents. They were validated for flow cytometry, Western blot, IF and/or IHC staining.
1.http://www.boster.com.cn/home/product/index.html?keywords=bm0002;
2.https://www.ptgcn.com/products/VIM-Antibody-10366-1-AP.htm;
3.https://www.ptgcn.com/products/FN1-Antibody-66042-1-Ig.htm
